# A multi-evidence computational and experimental strategy for *Silene nerimaniae*: combining phytochemical characterization, bioactivity assessment, and ADEFRank-HCT-116-based metabolite prioritization

**DOI:** 10.1039/d6md00624h

**Published:** 2026-09-03

**Authors:** Ahmet Erharman, Sakina Yagi, Abdullahi Ibrahim Uba, Filip Nikolić, Uroš Gašić, Mikail Acar, Nurettin Dogan, Ismail Koyuncu, Ismail Yapıcı, Ilhami Gulcin, Meng-Yao Li, Gunes Ak, Bengusu H. Akgul, Gokhan Zengin

**Affiliations:** a Department of Computer Engineering, Faculty of Technology, Selcuk University Konya Türkiye; b Department of Botany, Faculty of Science, University of Khartoum Khartoum Sudan; c Department of Biostatistics and Medical Informatics, Faculty of Medicine, İstinye University 34396 Istanbul Türkiye; d Department of Plant Physiology, Institute for Biological Research “Siniša Stanković”, National Institute of Republic of Serbia, University of Belgrade Bulevar Despota Stefana 142 11108 Belgrade Serbia filip.nikolic@ibiss.bg.ac.rs uros.gasic@ibiss.bg.ac.rs; e Tunceli Vocational School, Depertmant Plant and Animal Production, Munzur University Tunceli Türkiye; f Department of Medical Biochemistry, Faculty of Medicine, Harran University Sanliurfa 63290 Türkiye; g Gümüşhane University Central Research Laboratory Gümüşhane Türkiye; h Department of Chemistry, Science Faculty, Ataturk University Erzurum Türkiye; i Rectorate of Agri İbrahim Çeçen University 04100 Agri Türkiye; j State Key Laboratory of Systems Medicine for Cancer, Shanghai Cancer Institute, Renji Hospital, Shanghai Jiao Tong University School of Medicine Shanghai China limy@sioc.ac.cn; k Shanghai Key Laboratory for Cancer Systems Regulation and Clinical Translation, Shanghai Jiading District Central Hospital Shanghai China; l Department of Biology, Science Faculty, Selcuk University Konya Türkiye gokhanzengin@selcuk.edu.tr

## Abstract

This study presents the first comprehensive evaluation of the phytochemical composition, antioxidant properties, enzyme inhibitory effects, and cytotoxic potential of *Silene nerimaniae* G.E.Genç, Kandemir & İ.Genç (family Caryophyllaceae). Ethyl acetate, acetone, 70% acetone, and aqueous extracts were obtained from the aerial parts of the plant through maceration. Among the tested extracts, the acetone extract contained the highest total phenolic (24.37 mg GAE per g) and flavonoid (24.64 mg RE per g) contents. LC–MS analysis identified 52 bioactive constituents, including sugars, organic acids, phenolic acids, flavonoids, and fatty acids. The aqueous extract demonstrated the strongest radical scavenging activity against DPPH (23.02 mg TE per g) and ABTS (55.76 mg TE per g), as well as the highest Fe^3+^ reducing power (37.53 mg TE per g). In combination with the acetone extract, it also exhibited notable Cu^2+^ reducing activity (47.78 mg TE per g). Regarding enzyme inhibition, the ethyl acetate, acetone, and hydroacetone extracts displayed the strongest inhibitory activities against acetylcholinesterase (1.87 mg GALA per g), butyrylcholinesterase (2.92 mg GALA per g), and tyrosinase (60.44 mg KAE per g), respectively. All three extracts also inhibited α-amylase activity within the range of 0.73–0.77 mmol ACAE per g (*p* ≥ 0.05). In addition, the hydroacetone extract showed considerable inhibition of human carbonic anhydrase isoenzymes I (IC_50_ = 38.78 μg mL^−1^) and II (IC_50_ = 19.59 μg mL^−1^). The ethyl acetate extract exhibited the strongest cytotoxic effect against the colorectal cancer cell line HCT-116, with an IC_50_ value of 0.639 μg mL^−1^ and a selectivity index of 1.7. To contextualize this HCT-116-associated activity at the metabolite level, an ADEFRank-HCT116 (abundance–domain–evidence fusion ranking) workflow was applied by integrating EtOAc abundance, HCT-116-oriented QSAR evidence, applicability-domain confidence, and structure-assignment reliability. This analysis prioritized diosmetin, luteolin, eriodyctiol, acacetin, and isorhamnetin as core candidates for structure-based follow-up. Molecular docking screening predicted favorable docking profiles for the prioritized candidates, while 100 ns molecular dynamics simulations were used to assess the dynamic stability of the representative luteolin–PI3Kα, diosmetin–AKT1, and isorhamnetin–GSK-3β complexes. Taken together, these results indicate that *S. nerimaniae* may represent a valuable source of active compounds for various functional nutraceutical, cosmetic and pharmaceutical uses.

## Abstract 2

*Silene nerimaniae* may be considered as a valuable source of active compounds for various functional health-promoting applications.

## Graphical abstract

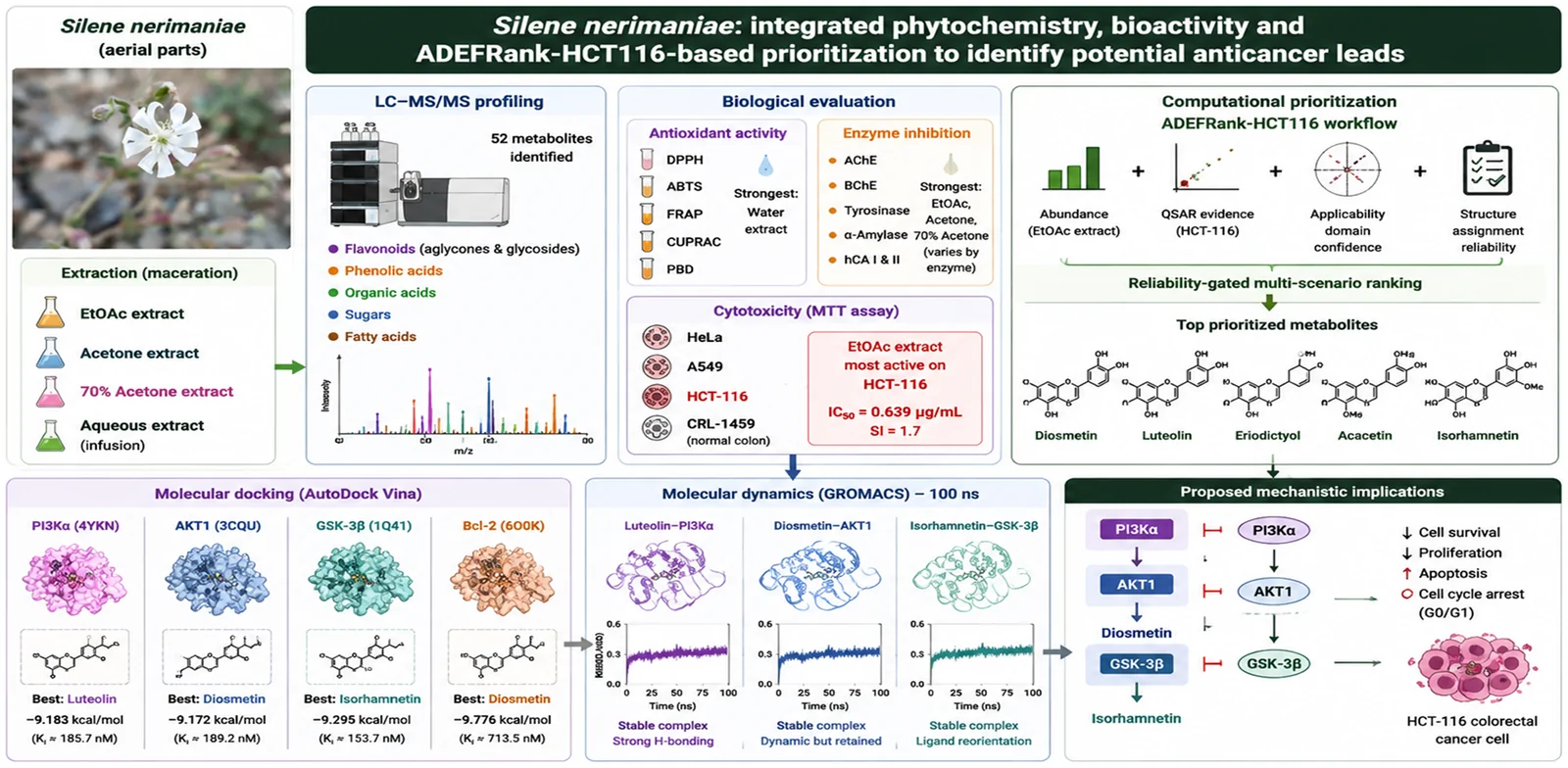

## Introduction

The genus *Silene* (family Caryophyllaceae) includes over 700 species that are predominantly found in the temperate regions of Eurasia and North America, with some species also occurring in Africa.^1^ Members of this genus are primarily herbaceous plants, although a few exist as small shrubs or subshrubs.^2^ In addition to containing several cultivated plants, the genus also includes many common weed species. Certain species, including *S. acaulis*, *S. multifida*, and *S. regia*, are grown ornamentally due to their attractive flowers. The roots of species such as *S. latifolia*, *S. acaulis*, *S. kumaonensis*, and *S. conoidea* have traditionally been utilized as natural soap substitutes.^3^ Some species are edible; for example, the young leaves and shoots of *S. vulgaris* are valued in the traditional cuisines of Turkey, Italy, Austria, and Spain.^4^ Various *Silene* species have also been employed in folk medicine for the treatment of inflammation, bronchitis, colds, and infections, as well as for their diuretic, antipyretic, analgesic, and emetic properties.^1,3^ Phytochemical studies of the genus have identified numerous bioactive compounds, including phytoecdysteroids (podecdysone C, sileneoside A, 2-deoxyecdysterone, *etc.*), triterpene saponins (jenisseensoside A, pachystegioside A, glanduloside C, *etc.*), terpenes (lutein, α-amyrin, β-carotene, *etc.*), flavonoids (apigenin, isovitexin, isoscoparin, homoorientin, *etc.*), anthocyanidins (idaein, kuromanin chloride, procyanidin B2, *etc.*), nitrogen-containing compounds (siliendine A, B, C, *etc.*), and sterols (stigmasterol glucoside, a-spinasterol, *etc.*).^1,3,5–7^ Furthermore, *Silene* species have been shown to exhibit diverse biological activities, such as antimicrobial, antifungal, antiviral, antioxidant, antitumor, immunomodulatory, adaptogenic, and actoprotective effects.^1,8–10^

In Turkey, the genus *Silene* is represented by 138 species classified into 31 sections, approximately 42% of which are endemic. One of these species, *Silene nerimaniae* G.E. Genç, Kandemir & İ. Genç, was described as a new taxon by Genç *et al.* in 2007 and is assigned to section *Auriculatae*. This species is endemic to Erzincan in Eastern Anatolia and belongs to the Irano–Turanian floristic region.^11^ Morphologically, *S. nerimaniae* is characterized as a densely tufted, suffruticose perennial with vegetative shoots bearing simple clustered leaves and flowers occurring solitarily or in 2–3-flowered dichasial cymes.^11^

Natural products remain an important source of pharmacologically relevant chemical scaffolds, but the biological interpretation of complex plant extracts is often limited by metabolite complexity, variable abundance, structural uncertainty, and the difficulty of assigning observed bioactivity to individual constituents.^12–14^ Recent advances in artificial intelligence, machine learning, cheminformatics, and computational drug-design workflows provide opportunities to complement natural-product profiling by predicting biological activity, prioritizing candidate metabolites, and generating target-level mechanistic hypotheses.^12,15^ In this context, we used an HCT-116-oriented abundance–domain–evidence fusion ranking workflow, hereafter referred to as ADEFRank-HCT116, to integrate extract-level metabolite abundance, QSAR-derived activity evidence, applicability-domain confidence, and structure-assignment reliability for prioritizing LC–MS-annotated metabolites.

To the best of our knowledge, no previous studies have investigated the phytochemical composition or biological activities of *S. nerimaniae*. Therefore, the present study aimed to examine the chemical profile and biological potential of extracts obtained from the aerial parts of the plant. The investigated biological activities included antioxidant, enzyme inhibitory, and cytotoxic effects. Antioxidant activity was evaluated using radical scavenging, metal chelating, and reducing power assays. Enzyme inhibitory effects were assessed against acetylcholinesterase, butyrylcholinesterase, tyrosinase, α-amylase, and human carbonic anhydrase isoenzymes I and II. In addition, cytotoxic activity was tested on HeLa, A549, and HCT-116 cancer cell lines, as well as on normal CRL-1459 cells. Finally, LC–MS-based metabolite annotation and biological activity testing were combined with ADEFRank-HCT116, molecular docking, and molecular dynamics simulations to prioritize HCT-116-relevant metabolites of *S. nerimaniae* and to provide hypothesis-level mechanistic support for the EtOAc-associated cytotoxic activity.

## Materials and methods

### Plant collection

Plant samples were collected in 2022 from Pülümür (between Kovuluk and Kocatepe villages, GPS coordinate: 39°25′21″N, 39°44′07″E, 1650 m), Tunceli, Turkey. Dr. Mikail Acar officially carried out the botanical identification. A reference specimen bearing the code MA-2066 was placed at the Munzur University herbarium. The aerial components were separated immediately after collection and allowed to air dry at room temperature in a well-ventilated, shady area. The plant material was pulverized into a fine powder once it had dried completely. The powdered samples were kept under constant conditions in light-proof containers to maintain chemical stability and prevent spoiling.

### Plant extract preparation

To separate bioactive chemicals, four different solvents with different polarities (ethyl acetate, acetone, acetone/water (70%) and water) were used. Ten grams of dried plant material were mixed with two hundred milliliters of each solvent. Depending on the type of solvent used, the extraction process was different. The plant material was macerated by soaking it in organic solvents for a whole day at room temperature. Meanwhile, a 15 minute hot-water infusion was used for water-based extraction. Following extraction, the organic solvent extracts were concentrated under low pressure using a rotary evaporator, the water extract was processed by freeze-drying. The extraction yields are given in Table 1.

**Table 1:** Extraction yields (%), total phenolic (TPC) and flavonoid (TFC) contents in extracts from *Silene nerimaniae* aerial parts^a^

Extracts	Extraction yields (%)	TPC (mg GAE per g)	TFC (mg RE per g)
EA	8.83	23.90 ± 0.16^ab^	21.47 ± 0.12^b^
Acetone	9.13	24.37 ± 1.43^a^	24.64 ± 0.07^a^
Acetone/water	8.34	22.20 ± 0.20^c^	12.90 ± 0.06^c^
Water	8.56	22.49 ± 0.27^c^	9.78 ± 0.05^d^

a Values are means ± SD of three measurements; means within each column with different letters (a–d) differ significantly (*p* ≤ 0.05); GAE, gallic acid equivalents; RE, rutin equivalents.

### Total phenolic and flavonoid contents

Total phenolic^16^ and total flavonoid^17^ contents in the extracts were determined using validated colorimetric methods, following established procedures. Calibration curves were prepared with gallic acid for phenolics and rutin for flavonoids, enabling the expression of the results as GAE and RE per gram, respectively.

### LC/MS experimental part

The chemical analysis of *Silene* extracts was conducted using LC/MS (Thermo Scientific™ Vanquish™ Core HPLC system) coupled to the Orbitrap Exploris 120 mass spectrometer (San Jose, CA, USA). All LC/MS parameters are explained in detail in Stojković *et al.*^18^ The obtained MS data were processed and analyzed using R Studio software (version 2023.09.1, build 494) using enviPick and xcms R packages.^19^ Identification of bioactive compounds was conducted based on their chromatographic behavior and HRMS/MS^2^ data, with comparisons made to standard compounds, when available, and literature data about *Silene* species, for tentative identification.^20–24^ The literature review was performed by searching the SciFinder database^25^ using suggested molecular formulas and keywords. Data acquisition was carried out using the Xcalibur® data system (Thermo Finnigan, San Jose, CA, USA).

To differentiate between samples, hierarchical cluster analysis (HCA) plots were constructed in Morpheus software,^26^ based on the Spearman method of cluster agglomeration, adopting the average linkage method.

### Antioxidant assays

A set of *in vitro* assays was employed to evaluate antioxidant activity, following the protocol described by ref. 27. Reducing power was determined using FRAP and CUPRAC methods, while radical scavenging capacity was assessed by DPPH and ABTS assays. The outcomes of these four tests were converted into Trolox equivalents and reported as mg TE per gram of dried extract. In addition, total antioxidant capacity was measured using the phosphomolybdenum (PBD) assay (mmol TE per g), and metal chelating activity was expressed as mg EDTA equivalents per gram (mg EDTAE per g). All experimental details are given in the SI.

### Enzyme inhibitory activity assays

Following established colorimetric methods,^27^ the extracts were evaluated for their ability to inhibit key enzymes: acetylcholinesterase (AChE), butyrylcholinesterase (BChE), tyrosinase, α-amylase, and human carbonic anhydrase isoenzymes I and II (hCA I and hCA II).^28^ To ensure comparability among samples, inhibition results were expressed relative to appropriate reference compounds. Accordingly, cholinesterase inhibition was reported as mg GALA per g, α-amylase inhibition as mmol ACAE per g, and tyrosinase inhibition as mg KAE per g. The inhibitory activity of human carbonic anhydrase isoenzymes I and II (hCA I and hCA II) was explained as IC_50_ values. All experimental details are given in the SI.

### Cell analysis

#### Cell culture

The following cancer and normal cell lines obtained from ATCC and stored in liquid nitrogen were used for the study. HeLa (serviks adenocarcinoma), A549 (lung adenocarcinoma), HCT-116 (colon adenocarcinoma) and CRL-1459 (colon normal) cells were cultured in DMEM-F12/RPMI-1640 media supplemented with 10% fetal bovine serum (FBS) and 100 μg ml^−1^ of streptomycin/100 IU mL^−1^ of penicillin in incubators at 37 °C under humid conditions containing 5% CO_2_.

#### Cell viability assay

The cytotoxic effects of the tested extracts were assessed using the MTT (3-(4,5-dimethylthiazol-2-yl)-2,5-diphenyltetrazolium bromide) assay. The cells (HeLa, A549, HCT-116 and CRL-1459) were incubated in 96 well sterile plates for 24 hours with 1 × 10^4^ cells per well. The media were removed and the extracts were incubated at different doses (2.5–200 μg mL^−1^) for 24 hours. 10 μL of MTT (0.5 mg mL^−1^) were added into each as the reactive agent. After 4 hours of incubation, the media were removed and substituted with 100 μL of DMSO, after which measurements were performed at OD_570_–OD_690_nm using a plate reader (Thermo Multiskan GO, Thermo, USA). Following these measurements, plots were formed and IC_50_ values were calculated.

#### Selective index of *Silene nerimaniae* extracts in cancer cells compared to control cells

In this study, the cytotoxic effects of the tested extracts on different cancer cells were evaluated, and IC_50_ values were determined. Specific indices were calculated by comparing the results with normal healthy cells and cancer cells. Specific indices were used to determine the specificity of plant extracts against cancer cells. In our study, the specific indices of eight different plant extracts were evaluated in three different cancer cell lines (HeLa, A549, and HCT-116).

#### Apoptotic effect of *Silene nerimaniae* ethyl acetate extract on HCT-116 cancer cells

The ethyl acetate extract (1 μg ml^−1^) applied to HCT-116 cells was used to detect apoptosis. After incubation, cells treated with the extract were washed with PBS and fixed with 70% ethanol. After fixation, cells were washed with distilled water and stained with acridine orange/ethidium bromide (Cat No./ID No: A6014-E1510) (Sigma Aldrich, Germany) working solution, and images were obtained under a fluorescence microscope.

#### Apoptotic effect of *Silene nerimaniae* ethyl acetate extract on HCT-116 cancer cells

The apoptotic effect of the substances was investigated. Commercial FITC Annexin V Apoptosis Detection kit I (BD Biosciences, New Jersey, USA) was applied according to the manufacturer's information protocol. HCT-116 cells were seeded in 6-well plates (5 × 105 cells per well). After 24 h of incubation, the extract (1 μg ml^−1^) was applied, followed by a 24 h incubation. Cells were grown using trypsin and transferred to new 1 × 106 inch tubes in 1× binding buffer. Each tube was incubated for 15 min at room temperature. Then, 5 μL of fluorochrome-conjugated Annexin V and 5 μL of propidium iodide dye were added. 100 μl of 1× binding buffer was added to the cells, centrifuged at 1200 rpm, and incubated for 5 min. Finally, the cells were analyzed by flow cytometry (BD, Via, New Jersey, USA).

#### Cell cycle analysis of *Silene nerimaniae* ethyl acetate extract on HCT-116 cancer cells

We used the BD Cycle-test kit (New Jersey, USA) containing PI dye to analyze the cell cycle. The protocol involved seeding 1–6105 cells per ml into 6-well sterile plates and applying the indicated concentration of substance 13 (1 μg ml^−1^) approximately 1 day later. The cells were incubated for another 24 hours. Cells were lifted with trypsin and centrifuged at 1500 rpm for 5 minutes, then the supernatant was removed and suspended. 1 ml of PBS was added to the cell suspensions and washed twice. 1× binding buffer (for binding of dyes) was added and the supernatant was removed by centrifugation at 1500 rpm for 5 minutes. 250 μl of solution A (Trypsin) was added to the cell pellet and incubated in the dark for 10 min with gentle mixing. Then, 200 μl of solution B (trypsin inhibitor) was added and incubated in the dark for 10 min. Cells were incubated with 200 μl of solution C (PI) for 10 min in the dark and then analyzed using a BD Facs via (BD, New Jersey, USA) flow cytometry instrument.

### Computational prioritization of annotated metabolites using ADEFRank-HCT116

#### QSAR model construction and applicability-domain assessment

The LC–MS-annotated metabolites of *S. nerimaniae* were computationally prioritized using ADEFRank-HCT116, a custom workflow designed to integrate extract-level metabolite abundance, HCT-116-oriented QSAR evidence, applicability-domain assessment, and structure-assignment confidence. The workflow was applied after LC–MS annotation and before structure-based molecular docking and molecular dynamics analyses.

The annotated metabolites were first curated according to the availability and reliability of their chemical structures. Compounds with sufficiently resolved structures and valid molecular representations were assigned to the core analysis set, whereas metabolites with unresolved positional, isomeric, or structural ambiguity were retained as a sensitivity-only set. This separation was used to distinguish structurally reliable metabolites from chemically relevant but structurally uncertain annotations during downstream prioritization.

ChEMBL-derived HCT-116 bioactivity records were curated and processed at the molecule level before QSAR modelling.^29^ Cell-based functional cytotoxicity and viability records associated with HCT-116 were standardized and converted to *p*_Activity_ values where applicable. Record-level activity data were aggregated by molecule using the median *p*_Activity_ value to reduce assay-level duplication. The primary training source was the molecule-level conservative non-conflict dataset, which contained 23 152 unique molecules with valid canonical SMILES and non-null *p*_Activity_ values. Records with conflicting activity annotations were excluded from the main training source. Molecular structures were processed using RDKit^30^ and compounds were represented using Morgan fingerprints.^31^

Two binary QSAR classification settings were generated. In the conservative p60 setting, compounds with *p*_Activity_ ≥ 6.0 were labelled as active and those with *p*_Activity_ < 5.0 as inactive, while the intermediate 5.0–6.0 range was omitted from model training. In the moderate p55 setting, compounds with *p*_Activity_ ≥ 5.5 were labelled as active and those with *p*_Activity_ < 5.0 as inactive, while the 5.0–5.5 gray zone was excluded. Thus, the p60 model used 8050 active and 6092 inactive molecules, whereas the p55 model used 11 961 active and 6092 inactive molecules. Gray-zone exclusion and molecule-level aggregation were used to reduce label ambiguity and avoid over-weighting repeated assay records.

Random forest classifiers were trained separately for the p60 and p55 datasets.^32,33^ Model performance was evaluated using both stratified random train/test splitting and Bemis–Murcko scaffold-based splitting.^34^ The random split was used to estimate within-distribution classification performance, whereas the scaffold split provided a more stringent assessment under chemical-series separation. In addition to ROC-AUC, PR-AUC/average precision, F1-score, Matthews correlation coefficient, balanced accuracy, sensitivity, specificity, and confusion-matrix counts were reported to provide a more complete evaluation of classifier behavior. Applicability-domain confidence for each *Silene* metabolite was estimated from its maximum Tanimoto similarity to the ChEMBL training compounds. This applicability-domain estimate was combined with structure-assignment confidence to define a reliability gate for downstream ranking. QSAR-derived probabilities were used only as HCT-116-oriented computational activity-evidence layers within ADEFRank and were not interpreted as direct experimental cytotoxicity values.

#### Reliability-gated ADEFRank ranking

For HCT-116-focused prioritization, EtOAc relative abundance was used as the abundance-evidence layer because the ethyl acetate extract showed the strongest HCT-116 cytotoxic profile among the tested extracts. QSAR outputs from the p55 and p60 models were converted into percentile-ranked activity-evidence layers and summarized as a QSAR evidence component. For each abundance/QSAR weighting scenario, the evidence score was calculated as:evidence_score_ = *w* × abundance_percentile_ + (1 − *w*) × QSAR_percentile_where *w* represents the abundance weight. A predefined grid of 39 abundance/QSAR weighting scenarios was evaluated to avoid relying on a single manually selected weight. The scenario-level score was then adjusted using a reliability gate:final_scenario_score__ = evidence_score_ × reliability_gate_The reliability gate was calculated as the geometric mean of applicability-domain confidence and structure-assignment confidence, so that compounds with uncertain structural assignment or lower similarity to the QSAR training domain were down-weighted during ranking. Candidate ranks were summarized across all weighting scenarios using median rank, top-ranking frequency, top-5 frequency, rank-IQR, and Pareto-front assignment. This multi-scenario strategy was used to identify metabolites showing stable support across alternative abundance- and QSAR-weighted conditions rather than relying on a single fixed-weight score. Robust rank aggregation and Pareto-front concepts were used only as cross-scenario ranking summaries and did not override the primary reliability-gated scoring procedure.^35,36^

#### Independent external evidence audit

External evidence was audited independently after ADEFRank-HCT116 prioritization and was not used to train the QSAR models or to modify ADEFRank scores. Candidate structures were searched against local ChEMBL HCT-116 activity records, NCI-60/CellMiner-derived evidence,^37^ and strictly curated PubChem BioAssay records.^38^ For the ChEMBL audit, exact InChIKey-level matches were distinguished from first-block connectivity-only matches, and candidate molecules overlapping with QSAR training molecules were flagged as training-overlap evidence rather than independent validation.

External evidence was classified as direct HCT-116 viability/cytotoxicity support, HCT116 cell-context mechanistic support, target-level mechanistic support, weak/negative contextual support, or no usable external evidence. These external resources were used only for post-ranking interpretation and did not override the primary reliability-gated ADEFRank-HCT116 hierarchy.

#### Molecular docking analysis of prioritized metabolites

Molecular docking was performed as a structure-based follow-up analysis of the primary ADEFRank-HCT116 core candidates. The docking ligand set included five prioritized metabolites, diosmetin, luteolin, eriodyctiol, acacetin, and isorhamnetin, against four HCT-116-associated protein targets: PI3Kα (PDB ID: 4YKN), AKT1 (PDB ID: 3CQU), GSK-3β (PDB ID: 1Q41), and Bcl-2 (PDB ID: 6O0K). Protein structures were obtained from the RCSB Protein Data Bank.^39,40^ Diosmin was retained in the complete ADEFRank output as a QSAR-supported low-abundance observation but was not included in the primary docking ligand set. The target panel was selected to provide a focused structure-based interrogation of signaling axes relevant to the HCT-116 phenotype observed for the EtOAc extract. Because the EtOAc extract showed the strongest HCT-116 cytotoxic profile together with apoptosis-associated changes and cell-cycle perturbation, the docking panel was restricted to representative proteins involved in cancer-cell survival, proliferation, kinase regulation, and apoptosis control. PI3Kα and AKT1 were included as central components of the PI3K/AKT survival and proliferation axis, GSK-3β was included as a kinase-regulatory protein linked to cell-cycle and survival signaling, and Bcl-2 was included as an anti-apoptotic regulator.^41–45^ In addition to biological relevance, the selected targets had experimentally resolved protein structures with ligand-defined binding pockets suitable for structure-based docking. External-evidence findings were used only for post-ranking contextual interpretation and were not used to automatically expand or redefine the docking target panel.

Ligand structures were prepared from curated SMILES representations. Three-dimensional conformers were generated using RDKit and energy-minimized with the MMFF94s force field.^46,47^ The optimized ligand structures were converted to PDBQT format using Meeko.^48^

Receptor structures were inspected before docking preparation. For each protein, the co-crystallized ligand was used to define the binding region and to support native ligand-based pocket checks, but it was removed before docking the *S. nerimaniae* metabolites. Crystallographic water molecules, buffer components, salts, and unrelated heteroatoms were removed unless they were required to define the ligand-binding environment. For multichain structures, the chain or chain assembly forming the ligand-defined binding pocket was retained, while unrelated chains or non-relevant structural components were excluded when applicable. Polar hydrogens, AutoDock-compatible atom types, and partial charges were assigned during receptor PDBQT preparation using Meeko. Native-ligand PDBQT conversion was supported by Open Babel when required.^49^ Prepared receptors were checked to ensure that the docking boxes were centered on the intended native-ligand-defined binding regions.

Docking grids were defined according to the corresponding co-crystallized ligand-binding regions, and grid centers and box dimensions are reported in Table S7. Docking calculations were performed using AutoDock Vina v1.2.7 with an exhaustiveness value of 32, 10 output modes, and an energy range of 4 kcal mol^−1^.^49–51^ For each ligand–target pair, 10 independent docking runs were conducted, and the best score as well as mean ± SD values were reported.

Native ligand-based checks were used to assess whether the docking setup reproduced or localized the intended binding pocket. Classical global redocking RMSD was used where appropriate, whereas local-pose geometry checks were reported for systems where global redocking was not used as the primary validation metric. Native-to-docked ligand centroid distances for *Silene* metabolites were evaluated separately as native-ligand-defined pocket proximity indicators and were not interpreted as redocking RMSD values. Estimated *K*_i_ values were calculated from Vina scores using *K*_i_(*M*) = exp(Δ*G*/*RT*), where Δ*G* is the Vina docking score in kcal mol^−1^, *R* is 0.001987 kcal mol^−1^ K^−1^, and *T* is 298.15 K; the resulting molar values were converted to nM. These *K*_i_ values were reported only as approximate docking-derived descriptors and were not treated as experimental binding constants.

Protein–ligand contact features were summarized using an in-house geometry-based Python analysis based on uniform distance/contact criteria across all complexes. The analysis included nearby residue counts, closest residue distances, and putative hydrogen-bond and hydrophobic-contact candidates. These interaction features were used as comparative geometric descriptors for pose annotation and MD-system selection and were not interpreted as experimentally validated residue-level interactions. Representative complexes for molecular dynamics simulations were selected from the docking screen based on target-level docking rank, native-pocket occupancy, interaction-profile interpretability, HCT-116 target relevance, and consistency with ADEFRank candidate roles. The selected MD systems were luteolin–PI3Kα, diosmetin–AKT1, and isorhamnetin–GSK-3β.

#### Molecular dynamics simulations

Molecular dynamics (MD) simulations were performed to examine the temporal stability and conformational behavior of the representative protein–ligand complexes selected after docking analysis: luteolin–PI3Kα, diosmetin–AKT1, and isorhamnetin–GSK-3β. Simulations were carried out using a GROMACS 2024.3 CUDA-enabled workflow.^52^ Protein topologies were generated with the AMBER99SB-ILDN force field^53^ while ligand topologies were prepared using ACPYPE/AmberTools with GAFF2 parameters and AM1-BCC partial charges.^54–56^

Each complex was solvated in a dodecahedral box using TIP3P-compatible water, maintaining a minimum solute–box distance of 1.0 nm, and the systems were neutralized with Na^+^/Cl^−^ ions to 0.15 M NaCl.^57^ Energy minimization was performed using the steepest descent algorithm until the maximum force was below 1000 kJ mol^−1^ nm^−1^. The systems were then equilibrated for 100 ps under NVT conditions and 100 ps under NPT conditions at 310 K and 1 bar. Temperature coupling was performed using the V-rescale thermostat,^58^ while pressure coupling was applied using C-rescale during equilibration and Parrinello–Rahman during production simulations.^59,60^

Production simulations were run for 100 ns with a 2 fs time step. Long-range electrostatics were treated using the particle mesh Ewald method,^61^ hydrogen-containing bonds were constrained with the LINCS algorithm,^62^ and periodic boundary conditions were applied in all directions. The resulting trajectories were analyzed to evaluate protein backbone RMSD, ligand RMSD, residue flexibility, radius of gyration, solvent-accessible surface area, hydrogen-bond persistence, minimum protein–ligand distance, and protein–ligand contact dynamics. RMSF was calculated using protein Cα atoms and plotted according to the sequential residue index to avoid chain- and residue-numbering discontinuities among the analyzed protein structures. These simulations were used as qualitative dynamic-support analyses of representative docking-derived complexes and were not designed to provide quantitative binding free-energy estimates.

#### Statistical analysis

All experiments were conducted in triplicate, and variations among extract samples were evaluated for statistical significance. Data were analyzed using one-way ANOVA (GraphPad Prism, version 9.2), followed by Tukey's *post hoc* test for pairwise group comparisons. A *p*-value below 0.05 was regarded as statistically significant.

## Results and discussion

### Chemical composition

The TPC and TFC of various extracts obtained from the aerial parts of *S. nerimaniae* were evaluated, and the findings are summarized in Table 1. TPC values ranged from 22.20 to 24.37 mg GAE per g, with the acetone and EtOAc extracts showing the highest levels, while the aqueous and hydroacetone extracts exhibited similar contents. TFC values varied between 9.78 and 24.64 mg RE per g. Among the extracts, the acetone and EtOAc extracts displayed respectively the highest content, whereas the aqueous extract contained the lowest amount. Furthermore, the comparison between TPC and TFC suggested that flavonoids were the predominant constituents of the extracts. This finding is consistent with previous studies conducted on other *Silene* species.^20^

Chemical analysis of *Silene* extracts led to the identification of 52 bioactive compounds. The structures of the compounds were proposed based on a review of the literature on *Silene* metabolites, where available. Table 2 lists the identified compounds, divided into several groups according to their basic chemical structures: sugars (3 compounds), organic acids (4 compounds), phenolic acids (10 compounds), flavonoid *C*,*O*-glycosides (6 compounds), flavonoid *C*-glycosides (6 compounds), flavonoid *O*-glycosides (9 compounds), flavonoid aglycones (9 compounds), and fatty acids (5 compounds). In the same table, there are also references that confirm the previous identification of certain compounds in some of *Silene* species. A major scientific contribution of this study is the identification of 13 compounds reported for the first time within the genus *Silene* (indicated as “NA” in the literature column).

**Table 2:** LC–MS data on metabolites identified in *Silene nerimaniae* extracts (*t*_R_ – retention time (min); Δ ppm – mean mass accuracy error; NA – not available)

No.	Compound name	*t*_R_, min	Molecular formula, [M–H]^−^	Calculated mass, *m*/*z*	Exact mass, *m*/*z*	Δ ppm	MS^2^ fragments, (% base peak)	Previously reported in *Silene*
*Sugars*								
1	Sucrose	0.46	C_12_H_21_O_11_^−^	341.10894	341.10802	2.68	59.01377(63), 71.01380(40), **89.02433**(100), 101.02433(30), 113.02437(25), 119.03492(35)	86
2	Glucose	0.47	C_6_H_11_O_6_^−^	179.05611	179.05595	0.88	**59.01375**(100), 71.01378(49), 85.02941(4), 89.02431(44), 101.02436(4), 113.02433(6)	87
3	Arabinose	0.48	C_5_H_9_O_5_^−^	149.04555	149.04539	1.07	**59.01375**(100), 89.02429(8), 149.04546(7)	88
*Organic acids*								
4	Galacturonic acid	0.48	C_6_H_11_O_7_^−^	195.05103	195.05080	1.14	**75.00871**(100), 87.00871(17), 99.00871(30), 129.01933(45), 159.02983(7), 177.04024(4)	88
5	Malic acid	0.49	C_4_H_5_O_5_^−^	133.01420	133.01419	0.09	71.01377(39), 72.99304(9), 89.02431(8), **115.00361**(100), 133.01418(47)	21
6	Citric acid	0.58	C_6_H_7_O_7_^−^	191.01973	191.01954	0.95	85.02941(35), 87.00867(50), 101.06064(4), **111.00867**(100), 129.01930(7), 191.01964(7)	21
7	Glutaric acid	0.72	C_5_H_7_O_4_^−^	131.03498	131.03491	0.58	**87.04513**(100), 113.02454(10), 131.03505(33)	21
*Phenolic acids*								
8	Vanillic acid hexoside	0.64	C_14_H_17_O_9_^−^	329.08781	329.08757	0.73	108.02164(14), 123.04510(33), 152.01131(19), **167.03491**(100)	NA
9	Gallic acid hexoside	0.64	C_13_H_15_O_10_^−^	331.06707	331.06689	0.55	125.02434(64), 151.00360(49), 168.00632(31), 169.01408(89), **331.06711**(100)	NA
10	Dihydroxybenzoic acid hexoside	0.65	C_13_H_15_O_9_^−^	315.07216	315.07204	0.35	108.02168(38), 109.02943(35), 152.01152(90), **153.01930**(100), 315.07278(31)	NA
11	Dihydroxybenzoic acid	0.82	C_7_H_5_O_4_^−^	153.01933	153.01923	0.67	**109.02946**(100), 153.01929(31)	20
12	Hydroxybenzoic acid hexoside	1.00	C_13_H_15_O_8_^−^	299.07724	299.07703	0.72	93.03450(21), **137.02429**(100)	NA
13	Hydroxybenzoic acid 1	1.28	C_7_H_5_O_3_^−^	137.02442	137.02434	0.59	93.03447(35), **137.02429**(100)	20
14	Coumaric acid hexoside	1.40	C_15_H_17_O_8_^−^	325.09289	325.09268	0.66	119.05014(54), 145.02943(11), **163.03996**(100)	NA
15	*p*-Coumaric acid	4.31	C_9_H_7_O_3_^−^	163.04007	163.03994	0.76	**119.05019**(100), 163.03999(12)	20
16	Ferulic acid	4.93	C_10_H_9_O_4_^−^	193.05063	193.05042	1.12	**134.03731**(100), 149.06079(21), 178.02710(57), 193.05051(18)	22
17	Hydroxybenzoic acid 2	5.60	C_7_H_5_O_3_^−^	137.02442	137.02441	0.07	93.03449(87), **137.02440**(100)	20
*Flavonoid C,O-glycosides*								
18	Apigenin 6-*C*-hexoside-7-*O*-hexoside (saponarin)	4.64	C_27_H_29_O_15_^−^	593.15119	593.15054	1.11	297.04028(36), **311.05585**(100), 341.06586(13), 431.09772(28), 473.10803(16), 593.15082(60)	89
19	Apigenin 6-*C*-(2″-pentosyl)-hexoside-7-*O*-hexoside	4.64	C_32_H_37_O_19_^−^	725.19345	725.19248	1.34	282.05267(4), **293.04523**(100), 311.05576(26), 323.05524(4), 341.06583(6), 413.08698(61), 443.09787(6), 563.14062(20), 605.14984(12)	90
20	Malonyl-apigenin 6-*C*-(2″-pentosyl)-hexoside-7-*O*-hexoside	4.90	C_35_H_39_O_22_^−^	811.19385	811.19239	1.80	**293.04529**(100), 311.05600(8), 323.05591(11), 353.06607(7), 413.08698(64), 605.15063(27)	NA
21	Apigenin 6-*C*-(2″-pentosyl)-hexoside-7-*O*-(6″-feruloyl)-hexoside	5.43	C_42_H_45_O_22_^−^	901.24080	901.23948	1.46	**293.04538**(100), 311.05582(16), 323.05563(10), 341.06604(16), 413.08713(63), 563.14001(33)	NA
22	Malonyl-apigenin 6-*C*-(2″-pentosyl)-hexoside-7-*O*-(6″-feruloyl)-hexoside	5.50	C_45_H_47_O_25_^−^	987.24119	987.23987	1.33	**293.04523**(100), 311.05621(8), 323.05511(10), 335.05542(4), 341.06561(8), 353.06497(6), 413.08682(56), 485.10901(7), 605.15033(24)	NA
23	Apigenin 6-*C*-(2″-pentosyl)-hexoside-7-*O*-(6″-dihydroferuloyl)-hexoside	5.55	C_42_H_47_O_22_^−^	903.25645	903.25480	1.82	**293.04526**(100), 311.05569(15), 323.05569(10), 341.06586(17), 353.06552(7), 413.08688(65), 431.09756(3), 443.09790(5), 563.13995(35)	91
*Flavonoid C-glycosides*								
24	Luteolin 6-*C*-hexoside-8-*C*-pentoside (carlinoside)	4.75	C_26_H_27_O_15_^−^	579.13554	579.13478	1.32	298.04788(90), 309.04025(46), 327.05069(39), 338.04297(18), 339.04993(19), 357.06079(39), 369.06058(9), 429.08206(24), **459.09268**(100)	92
25	Luteolin 6-*C*-hexoside (isoorientin)	4.76	C_21_H_19_O_11_^−^	447.09329	447.09232	2.16	297.04037(12), 298.04803(4), 299.05554(8), **327.05063**(100), 357.06085(72)	92
26	Apigenin 6-*C*-(2″-pentosyl)-hexoside	5.11	C_26_H_27_O_14_^−^	563.14063	563.13980	1.47	131.03485(67), 149.04558(75), **293.04559**(100), 311.05463(20), 353.06619(22), 413.08606(30)	92
27	Apigenin 6-*C*-hexoside (isovitexin)	5.19	C_21_H_19_O_10_^−^	431.09837	431.09732	2.44	283.06113(13), **311.05591**(100), 323.05597(7), 341.06631(43), 431.09891(4)	92
28	Malonyl-apigenin 6-*C*-(2″-pentosyl)-hexoside	5.27	C_29_H_29_O_17_^−^	649.14102	649.14101	0.02	**293.04517**(100), 311.05582(10), 323.05545(11), 341.06580(8), 353.06537(5), 413.08694(22)	NA
29	Chrysoeriol 6-*C*-hexoside (Isoscoparin)	5.32	C_22_H_21_O_11_^−^	461.10894	461.10788	2.28	270.01434(4), 298.04843(27), 313.07227(6), **341.06607**(100), 353.06879(5), 371.07715(33)	93
*Flavonoid O-glycosides*								
30	Quercetin 3-*O*-(6″-rhamnosyl)-hexoside (rutin)	5.03	C_27_H_29_O_16_^−^	609.14611	609.14520	1.50	151.00388(2), 178.99854(2), 271.02374(2), **300.02713**(100), 301.03445(15), 609.14484(6)	23
31	Quercetin 3-*O*-hexoside (hyperoside)	5.26	C_21_H_19_O_12_^−^	463.08820	463.08725	2.06	151.00404(3), 178.99901(2), 255.03206(2), 271.02457(3), **300.02731**(100), 301.03494(39)	22
32	Kaempferol 3-*O*-(6″-rhamnosyl)-hexoside (nicotiflorin)	5.29	C_27_H_29_O_15_^−^	593.15119	593.15041	1.31	151.04002(3), 284.03235(56), 285.04016(67), **593.15076**(100)	22
33	Kaempferol 3-*O*-hexoside (astragalin)	5.58	C_21_H_19_O_11_^−^	447.09329	447.09225	2.31	227.03445(6), 255.02992(12), 256.03763(4), **284.03253**(100), 285.04004(29), 447.09433(18)	23
34	Apigenin 7-*O*-(6″-rhamnosyl)-hexoside (isorhoifolin)	5.65	C_27_H_29_O_14_^−^	577.15628	577.15562	1.14	268.03741(8), **269.0452**(100), 577.15491(9)	94
35	Apigenin 7-*O*-hexoside (apigenitrin)	5.69	C_21_H_19_O_10_^−^	431.09837	431.09738	2.30	268.03732(98), 269.04541(63), **431.09738**(100)	94
36	Diosmetin 7-*O*-(6″-rhamnosyl)-hexoside (diosmin)	5.72	C_28_H_31_O_15_^−^	607.16684	607.16618	1.10	283.02527(4), 284.03214(13), 298.04764(9), **299.05576**(100)	95
37	Luteolin 7-*O*-hexoside (cynaroside)	5.86	C_21_H_19_O_11_^−^	447.09329	447.09236	2.07	**285.04004**(100)	5
38	Rhamnetin 3-*O*-hexoside	6.36	C_22_H_21_O_12_^−^	477.10385	477.10273	2.35	165.01936(3), 299.01944(15), **314.04285**(100), 315.05051(39)	NA
*Flavonoid aglycones*								
39	Taxifolin	5.12	C_15_H_11_O_7_^−^	303.05103	303.05067	1.18	**125.02435**(100), 151.00337(8), 175.04018(18), 178.99901(9), 241.05156(11), 285.04016(35)	NA
40	Luteolin	6.41	C_15_H_9_O_6_^−^	285.04046	285.04007	1.36	151.00351(2), 175.04045(2), 241.04926(2), **285.04007**(100)	20
41	Eriodyctiol	6.41	C_15_H_11_O_6_^−^	287.05614	287.05522	3.22	107.01372(8), 125.02441(4), 135.04501(71), **151.00351**(100)	NA
42	Isorhamnetin	6.61	C_16_H_11_O_7_^−^	315.05103	315.05072	0.97	255.03029(5), 271.02457(8), **300.02747**(100)	NA
43	Naringenin	6.84	C_15_H_11_O_5_^−^	271.06120	271.06073	1.71	93.03454(11), 107.01375(10), 119.05018(39), **151.00357**(100), 177.01923(12), 271.06009(60)	20
44	Kaempferol	6.85	C_15_H_9_O_6_^−^	285.04046	285.04014	1.12	151.00359(2), **285.0401**(100)	20
45	Diosmetin	7.47	C_16_H_11_O_6_^−^	299.05611	299.05589	0.74	271.02460(9), 284.03238(61), **299.05588**(100)	95
46	Rhamnetin	7.48	C_16_H_11_O_7_^−^	315.05103	315.05074	0.90	121.02947(25), **165.01923**(100), 193.01419(16), 287.05521(7), 300.02728(26), 315.04965(51)	23
47	Acacetin	7.98	C_16_H_11_O_5_^−^	283.06120	283.06085	1.23	255.02965(3), **268.03723**(100), 283.06061(85)	22
*Fatty acids*								
48	Trihydroxyoctadecadienoic acid	7.01	C_18_H_31_O_5_^−^	327.21770	327.21741	0.87	171.10254(47), 183.13885(24), **211.13376**(100), 229.14433(70), 291.19638(19), 309.20636(5)	NA
49	Hydroxyoctadecatrienoic acid	9.18	C_18_H_29_O_3_^−^	293.21222	293.21191	1.05	**223.17014**(100), 235.16934(8), 249.18596(13), 275.20178(24), 293.21274(43)	NA
50	Hydroxyoctadecadienoic acid	10.22	C_18_H_31_O_3_^−^	295.22787	295.22750	1.25	**295.22733**(100)	NA
51	Hydroxyoctadecanoic acid	10.53	C_18_H_35_O_3_^−^	299.25917	299.25863	1.79	253.25357(2), 281.24811(2), **299.25885**(100)	96
52	Hydroxyeicosanoic acid	11.15	C_20_H_39_O_3_^−^	327.29047	327.29014	1.02	281.28442(2), 309.27921(2), **327.28998**(100)	96

Highly polar primary metabolites were eluted rapidly during the initial phase of the chromatographic run. Among carbohydrates, sucrose, glucose, and arabinose were annotated. Their corresponding MS^2^ spectra revealed characteristic product ions at *m*/*z* 59, 71, and 89, which originate from cross-ring cleavages of the sugar rings.

The simultaneous presence of galacturonic, malic, citric and glutaric acids confirms a highly active tricarboxylic acid (TCA) cycle in *S. nerimaniae*. These organic acid findings are in perfect agreement with established metabolic data documented for related species within the genus *Silene*.^21^

A total of 10 compounds were classified as phenolic acids or their derivatives. MS^2^ fragmentation of these glycosylated species consistently yielded a primary neutral loss of a hexose moiety (162 Da). This elimination frees the corresponding aglycone core as the dominant base peak (*e.g.*, the fragment ion at *m*/*z* 167 for vanillic acid or *m*/*z* 163 for coumaric acid). In contrast, free phenolic acids such as *p*-coumaric and ferulic acids displayed delayed elution (4.31 and 4.93 min, respectively) due to their reduced overall polarity compared to their glycosylated counterparts.

The structural elucidation of four newly reported apigenin derivatives within the genus *Silene* (compounds 20–22, and 28) was achieved through high-resolution collision-induced dissociation (CID). These specialized metabolites represent intricate carbohydrate architectures characterized by malonyl (86 Da) and feruloyl (176 Da) acylations, combined with both *C*- and *O*-glycosidic linkages.

For compound 20, the initial MS^2^ steps involve a competitive pathway between the neutral loss of the 7-*O*-linked hexose (162 Da) and the diagnostic loss of the malonyl unit as a combined neutral departure of carbon dioxide (CO_2_, 44 Da) and ketene (C_2_H_2_O, 42 Da), inducing an overall net reduction of 86 Da. The appearance of a prominent product ion at *m*/*z* 413 (64% of MS^2^ base peak) corresponds to the mono-glycosylated apigenin core structure. The final, highly stable MS^2^ base peak at *m*/*z* 293 was generated through the characteristic cross-ring cleavage of the remaining 6-*C*-hexosyl ring *via* the neutral loss of 120 Da. Compound 28 undergoes a highly parallel fragmentation process but lacks the 7-*O*-hexoside anchor. Its precursor ion (*m*/*z* 649) undergoes direct elimination of the malonyl unit (loss of 86 Da) to yield an intermediate at *m*/*z* 563, which subsequently collapses *via* internal cross-ring breakdown of the *C*-hexoside to yield the identical dominant MS^2^ base peak at *m*/*z* 293. In compound 21, CID triggers the fragmentation of the ester bond linking the ferulic acid, resulting in the neutral loss of the feruloyl–hexoside chain (338 Da; a combined loss of a feruloyl unit (176 Da) and a hexose residue (162 Da)). This fragmentation yields a key diagnostic intermediate ion at *m*/*z* 563 (33% of MS^2^ base peak). Further cross-ring cleavages of the remaining *C*-linked pentosyl–hexoside infrastructure yield the fragment at *m*/*z* 413 (63% of MS^2^ base peak), culminating in the generation of the aglycone-related MS^2^ base peak at *m*/*z* 293. Compound 22 introduces simultaneous dual acylation (both malonyl and feruloyl groups). Its tandem mass spectrum maps out a sequential cascade of neutral losses. The terminal malonyl group is readily cleaved (loss of 86 Da), occurring alongside the loss of the feruloyl–hexoside complex. This dual arrangement is verified by the product ion at *m*/*z* 605 (24% of MS^2^ base peak), which successfully retains the malonyl modification despite losing the feruloyl chain. Just like its structural analogs, the core matrix eventually resolves down to the fingerprint fragments at *m*/*z* 413 (56% of MS^2^ base peak) and the final MS^2^ base peak at *m*/*z* 293. The fact that all four novel compounds (20, 21, 22, and 28) ultimately resolve into the matching MS^2^ base peak at *m*/*z* 293 provides definitive proof that they share a unified structural core: an apigenin backbone substituted with a 6-*C*-linked sugar chain. Differences in their initial molecular weights are purely dictated by highly specific esterification and secondary *O*-glycosylation.

Identified flavonoid *O*-glycosides encompass rutin, hyperoside, nicotiflorin, and astragalin. Their tandem MS^2^ spectra are remarkably clean and simple with the MS^2^ base peak consistently reflecting the aglycone fragment. Between the retention time window of 5.12 and 7.98 min, 9 free flavonoid aglycones were successfully annotated. Among them, taxifolin, eriodictyol, isorhamnetin, and rhamnetin were for the first time reported in the genus *Silene*.

Eluting in the hydrophobic, late phase of the chromatogram are several polyhydroxylated and hydroxylated C18 and C20 fatty acids. Trihydroxyoctadecadienoic acid (compound 48) and hydroxyoctadecatrienoic acid (compound 49) stand out as brand-new additions to the chemical registry of the *Silene* genus. Meanwhile, hydroxyoctadecanoic and hydroxyeicosanoic acids are well-aligned with lipid data from related species. These oxylipin metabolites are known to fulfill critical roles in plant defense systems and stress-response signaling.

In order to compare the distribution of identified compounds through the given extraction solvents, hierarchical cluster analysis (HCA) was performed on peak area values (Table S1), obtained from full scan MS spectra. Fig. 1 clearly shows the distribution of compounds by polarity.

**Fig. 1:**
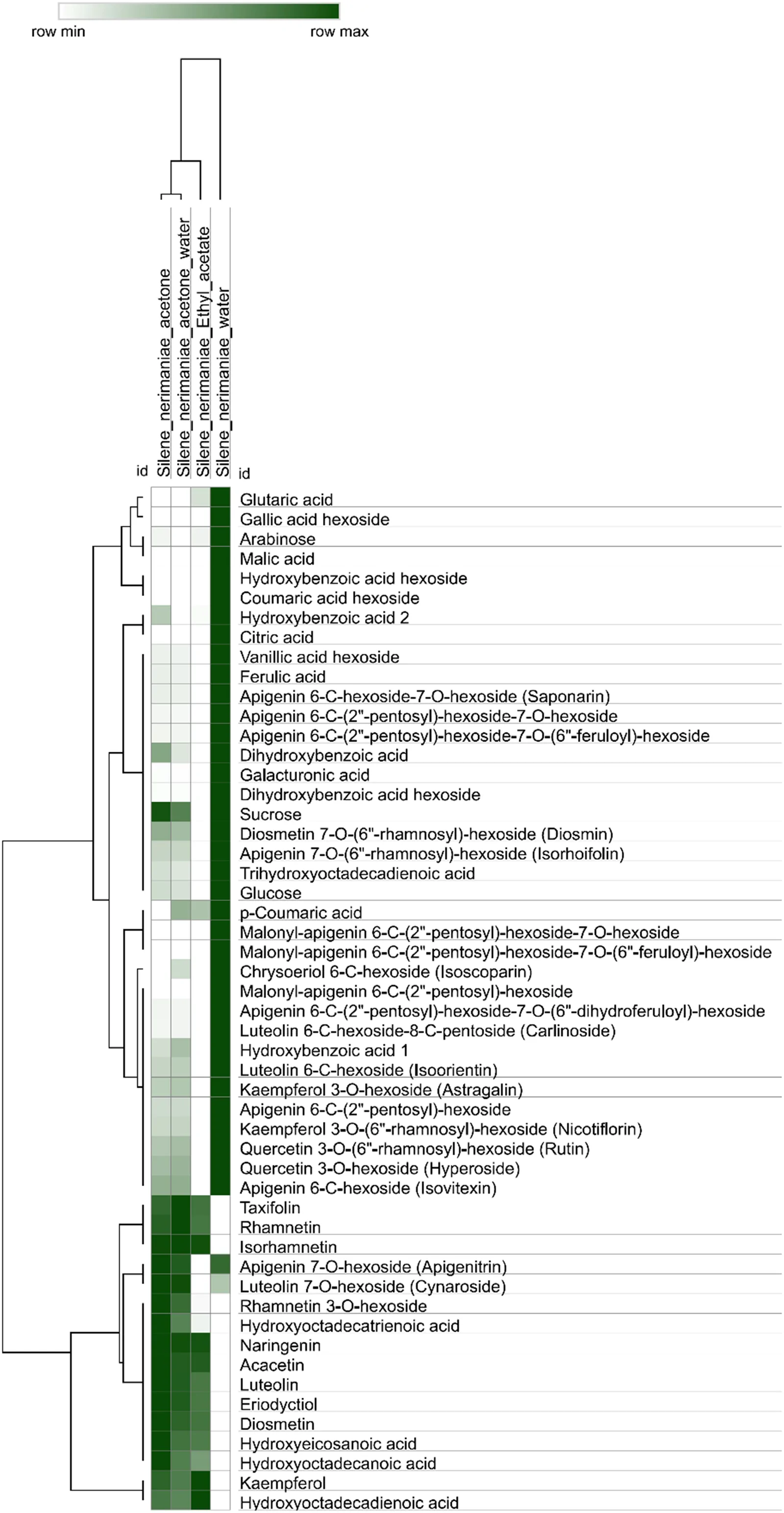
Heatmap of the scaled data of untargeted metabolite analysis, with the samples (both columns and rows) arranged according to the HCA (Spearman method of cluster agglomeration). The intensity of the green color indicates the abundance of the compounds in the samples, as shown in the color scale.

### Antioxidant activity

The antioxidant activity of different extracts obtained from the aerial parts of *S. nerimaniae* was assessed, and the results are summarized in Table 3. Among the tested extracts, only the aqueous extract exhibited notable radical scavenging activity in both assays (DPPH = 23.02 mg TE per g and ABTS = 55.76 mg TE per g), whereas the other extracts showed much lower values (≤ 6.64 mg TE per g). The cupric ion reducing capacity ranged from 42.35 to 47.78 mg TE per g, with the acetone and aqueous extracts demonstrating the highest and comparable activities (*p* ≥ 0.05). The EtOAc and hydroacetone extracts also presented similar, though slightly lower, values. The ferric ion reducing capacity varied between 19.24 and 37.53 mg TE per g, with the aqueous extract showing the strongest effect, followed by the acetone and hydroacetone extracts (*p* ≥ 0.05). Metal chelating activity ranged from 3.05 to 12.01 mg EDTAE per g, where the acetone extract exhibited the highest activity, while the hydroacetone extract showed the lowest. In terms of total antioxidant capacity, the EtOAc and acetone extracts displayed the highest values (0.90 and 0.91 mmol TE per g, respectively; (*p* ≥ 0.05)), whereas the aqueous extract demonstrated the lowest activity (0.61 mmol TE per g). Overall, it was observed that the aqueous extract, despite having the lowest total phenolic content (TPC), demonstrated the strongest antiradical and ion-reducing activities. In contrast, the acetone extract, which had the highest TPC and TFC, showed the weakest antiradical activity but exhibited superior Cu^2+^ reducing capacity, comparable to that of the aqueous extract. Additionally, it displayed the highest metal-chelating ability and total antioxidant activity. These findings indicate that phenolic bioactive compounds present in the various extracts possess different antioxidant capacities depending on the assay mechanism employed. Indeed, the use of multiple antioxidant assays has been recommended to provide a more comprehensive evaluation of bioactive potential, as each method offers complementary insights.^63^ Furthermore, analysis of the distribution of the identified compounds, particularly phenolic acids and flavonoids, revealed that these constituents were predominantly concentrated in the aqueous and acetone extracts, which may explain their remarkable antioxidant activity.^64,65^ Comparison with previous investigations on other *Silene* species, including *S. alba*, *S. conoidea*, *S. dichotoma*, *S. italica*, *S. supina*, and *S. vulgaris*, showed that those species exhibited stronger antioxidant effects than those observed in the present study, as the reported assays were conducted using methanolic extracts.^20^ Therefore, future studies should evaluate the antioxidant potential of *S. nerimaniae* using more polar organic solvents such as methanol and ethanol.

**Table 3:** Antioxidant properties of extracts from *Silene nerimaniae* aerial parts^a^

Extracts	DPPH^b^ (mg TE per g)	ABTS^b^ (mg TE per g)	CUPRAC^b^ (mg TE per g)	FRAP^b^ (mg TE per g)	MC^c^ (mg EDTAE per g)	PBD^d^ (mmol TE per g)
EA	2.47 ± 0.21^b^	5.24 ± 0.65^c^	42.44 ± 0.87^b^	19.24 ± 0.68^c^	9.49 ± 0.81^b^	0.90 ± 0.01^a^
Acetone	2.14 ± 0.19^c^	5.51 ± 0.49^c^	47.78 ± 3.43^a^	24.45 ± 0.84^b^	12.01 ± 0.15^a^	0.91 ± 0.05^a^
Acetone/water	2.53 ± 0.17^b^	6.64 ± 0.11^b^	42.35 ± 1.12^b^	23.40 ± 0.21^b^	3.05 ± 0.13^c^	0.82 ± 0.01^b^
Water	23.02 ± 0.25^a^	55.76 ± 0.20^a^	46.29 ± 0.86^a^	37.53 ± 0.79^a^	9.72 ± 0.26^b^	0.61 ± 0.07^c^

a Values are means ± SD of three measurements. Means within each column with different letters (a–d) differ significantly (*p* ≤ 0.05).

b mg Trolox equivalents per g of extract.

c mg EDTAE per g of extract.

d mmol Trolox equivalents per g of extract.

### Enzyme inhibition activity

Enzyme inhibition has been a very successful approach in drug discovery as it is often essential to selectively block key enzymes to control diseases that are still widespread throughout the world.^66^ This is nicely illustrated by therapy for Alzheimer's disease where inhibition of acetylcholinesterase increases the concentration of acetylcholine in the brain and hence helps to maintain cognitive performance.^67^ Similar logic is used in the case of diabetes treatment to control the level of blood glucose after eating foods rich in carbohydrates by inhibiting amylase and glucosidase activity.^68^ Also important is tyrosinase inhibition especially for the treatment of hyperpigmentation, since this enzyme is a central catalyst in melanin production.^69^ Synthetic inhibitors have been developed for all of these enzymatic targets, but safety concerns are often raised with their long-term use. These limitations have rekindled interest in natural compounds that could provide similar therapeutic benefits with improved safety. Thus, in the present study, we performed a series of enzyme inhibition assays along with broader bioactivity evaluations to determine the functional and pharmacological properties of the tested extracts.

The enzyme inhibitory potential of extracts derived from the aerial parts of *S. nerimaniae* was assessed against acetylcholinesterase (AChE), butyrylcholinesterase (BChE), tyrosinase (Tyr), and α-amylase, and the results are presented in Table 4. The aqueous extract exhibited the weakest activity against all four enzymes. The highest inhibitory effects against AChE (1.87 mg GALA per g), BChE (2.92 mg GALA per g), and tyrosinase (60.44 mg KAE per g) were observed for the EtOAc, acetone, and hydroacetone extracts, respectively. In contrast, the three extracts showed no significant differences in their inhibitory activity against α-amylase (0.73–0.77 mmol ACAE per g; *p* ≥ 0.05).

**Table 4:** Enzyme inhibitory properties of extracts from *Silene nerimaniae* aerial parts^a^

Extracts	AChE^b^ (mg GALA per g)	BChE^b^ (mg GALA per g)	Tyrosinase^c^ (mg KAE per g)	α-Amylase^d^ (mmol ACAE per g)
EA	1.87 ± 0.19^a^	2.73 ± 0.61^b^	56.17 ± 1.26^b^	0.76 ± 0.04^a^
Acetone	1.66 ± 0.08^b^	2.92 ± 0.05^a^	55.80 ± 2.20^b^	0.77 ± 0.03^a^
Acetone/water	1.35 ± 0.14^c^	1.70 ± 0.10^c^	60.44 ± 0.73^a^	0.73 ± 0.01^a^
Water	0.47 ± 0.06^d^	NA	37.74 ± 0.09^c^	0.08 ± 0.01^b^

a Values are means ± SD of three measurements. Means within each column with different letters (a–d) differ significantly (*p* ≤ 0.05).

b mg galantamine equivalents per g of extract.

c mg kojic acid per g of extract.

d mmol acarbose equivalents per g of extract.

Extracts were further evaluated for their inhibitory activity against human carbonic anhydrase isoenzymes hCA I and hCA II (Table 5). All tested extracts exhibited stronger inhibition of both isoenzymes compared to the standard control, acetazolamide (IC_50_ values: hCA I = 82.50 μg mL^−1^; hCA II = 116.00 μg mL^−1^), as indicated by their lower IC_50_ values. The extracts showed greater potency against hCA II, with the activity ranked as follows: hydroacetone (IC_50_ = 19.59 μg mL^−1^) > H_2_O (IC_50_ = 21.74 μg mL^−1^) > EtOAc (IC_50_ = 22.00 μg mL^−1^) > acetone (IC_50_ = 41.16 μg mL^−1^). For hCA I inhibition, the ranking was: hydroacetone (IC_50_ = 38.78 μg mL^−1^) > EtOAc (IC_50_ = 50.92 μg mL^−1^) > acetone (IC_50_ = 56.58 μg mL^−1^) > H_2_O (IC_50_ = 61.12 μg mL^−1^). However, some of the identified compounds in the present study were reported to possess enzyme inhibition potential. For example, rutin, gallic acid, ferulic acid, and naringenin have been reported to inhibit tyrosinase.^70–73^ In addition, rutin has also been shown to inhibit acetylcholinesterase.^74^*p*-Hydroxybenzoic acid, ferulic acid, and quercetin, are known to inhibit carbonic anhydrase.^75^

**Table 5:** hCA isoenzyme inhibition of *Silene nerimaniae* aerial part extracts. Results were expressed as IC_50_ (μg mL^−1^). AZA; acetazolamide

Extract name	hCA I		hCA II	
	IC_50_	*r* ^2^	IC_50_	*r* ^2^
EtOAc	50.92 ± 2.52	0.9935	22.00 ± 6.68	0.9663
Acetone	56.58 ± 1.77	0.9966	41.16 ± 5.80	0.9602
Acetone/water	38.78 ± 5.81	0.9771	19.59 ± 5.74	0.9707
Water	61.12 ± 1.21	0.9983	21.74 ± 3.70	0.9863
AZA	82.50 ± 3.75	0.9850	116.00 ± 4.56	0.9683

Previous studies have reported the enzyme inhibitory potential of some *Silene* species, including *S. alba*, *S. conoidea*, *S. dichotoma*, *S. italica*, *S. supina*, and *S. vulgaris*. These species demonstrated stronger anti-AChE activity (1.7–2.0 mg GALA per g) when methanol was used as the extraction solvent, compared with the activity observed for *S. nerimaniae* in the current study. Nevertheless, *S. nerimaniae* exhibited comparatively greater anti-BChE activity, whereas among the previously studied species, only *S. alba* showed notable anti-BChE potential (1.0 mg GALA per g).^20^ In addition, *S. nerimaniae* displayed higher anti-tyrosinase activity than the six reported species. These differences may be attributed not only to species variation but also to the extraction solvent employed, since the earlier investigations were conducted using methanolic extracts, while the present study evaluated four different extracts excluding methanol.^76^

### Cytotoxicity

The cytotoxic activity of extracts derived from the aerial parts of *S. nerimaniae* was assessed against cervical adenocarcinoma (HeLa), human lung carcinoma (A549), and colorectal cancer (HCT-116) cell lines, as well as non-cancerous human colon (CRL-1459) cells. The results are summarized in Table 6. All the extracts, with the exception of the aqueous extract, demonstrated strong toxicity toward the three cancer cell lines, but also affected the normal CRL-1459 cells. Among them, the EtOAc extract showed the most potent activity, with IC_50_ values of 0.639 μg mL^−1^ for HCT-116, 1.9 μg mL^−1^ for HeLa, and 2.34 μg mL^−1^ for A549. The remaining extracts exhibited IC_50_ values ranging from 10.20 to 16.73 μg mL^−1^ across the cancer cell lines. However, evaluation of the selectivity index (SI) revealed values below 1 for all the extracts, except for the EtOAc extract against HCT-116 cells, which showed an SI value of 1.7 (Table 7). The ethyl acetate extract's apoptotic activity was determined in HCT-116 colorectal carcinoma cells by AO/EB fluorescence staining and Annexin V/PI flow cytometry. The untreated control group showed 96.9% cell viability with early apoptotic, late apoptotic and necrotic cell populations of 1.0%, 1.6% and 0.5% of total, respectively. The extract (1 μg ml^−1^) significantly decreased viability to 48.7% with significant increases in the early apoptotic (9.1%), late apoptotic (21.2%) and necrotic (21.0%) fractions. These results indicate that the ethyl acetate extract induces cell death in HCT-116 cells through apoptotic and necrotic mechanisms in a substantial proportion of the cell population (Fig. 2). To further elucidate the antiproliferative mode of action of the extract, cell cycle distribution was analysed by propidium iodide staining and flow cytometry. In untreated HCT-116 cells 37.4% were in the G0/G1 phase, 27.3% were in the S phase and 28.5% were in the G2/M phase. The G0/G1 fraction increased to 51.3% and the populations of the S-phase and G2/M-phase decreased to 26.6% and 18.2%, respectively, after treatment of the extract (1 μg ml^−1^). The accumulation of G0/G1 phase during this transition indicates that the ethyl acetate extract is responsible for this.

**Table 6:** Cytotoxic effects of *Silene nerimaniae* aerial part extracts on cancer and normal cell lines (IC_50_)

Extracts	HeLa	A549	HCT-116	CRL-1459
EtOAc	1.9	2.34	0.639	1.09
Acetone	12.38	10.20	14.57	1.27
Acetone/water	16.73	10.57	10.93	1.17
Water	157.25	164.74	152.95	148.54

**Table 7:** Selective index evaluation of cancerous cells compared to CRL-1459 normal cells of the tested extracts

Cells	HeLa	A549	HCT-116
EtOAc	0.6	0.5	1.7
Acetone	0.1	0.1	0.1
Acetone/water	0.1	0.1	0.1
Water	0.9	0.9	1.0

**Fig. 2:**
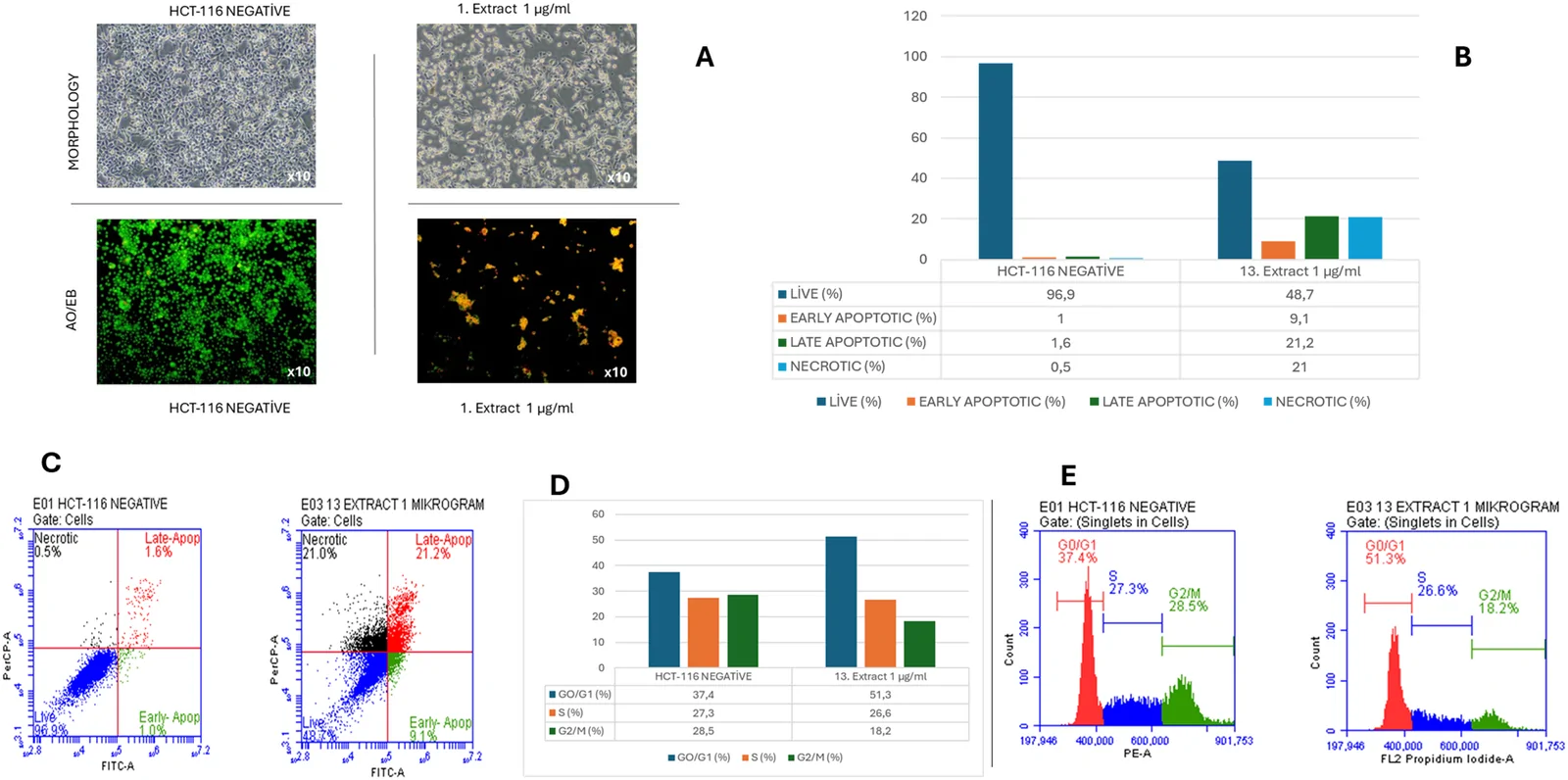
(A) AO/EB staining after the ethyl acetate extract (1 μg ml^−1^) was applied to HCT-116 cells. (B) Annexin-V/PI staining results after applying the ethyl acetate extract (1 μg ml^−1^) to HCT-116 cells. (C) Annexin-V/PI staining results after the ethyl acetate extract (1 μg ml^−1^) was applied to HCT-116 cells. Blue: live-[(FITC^−^)/(PI^−^)]; green: early apoptotic [(FITC^+^)/(PI^−^)]; red: late apoptotic [(FITC^+^)/(PI^+^)]; black: shows necrotic [(FITC^+^)/(PI^+^)] cells. (D) Cell cycle staining results after applying the ethyl acetate extract (1 μg ml^−1^) to HCT-116 cells. Red: G0/G1 phase; blue: S phase; green: G2/M phase. (E) Cell cycle staining results after applying the ethyl acetate extract (1 μg ml^−1^) to HCT-116 cells. Red: G0/G1 phase; blue: S phase; green: G2/M phase.

In the literature, several researchers have examined the cytotoxic effects of some *Silene* species. For example, Mamadalieva *et al.*^77^ determined the cytotoxic effects of *S. guntensis* extracts and the chloroform extract exhibited a potent cytotoxic effect on HeLa (IC_50_: 25.58 μg ml^−1^), HepG-2 (IC_50_: 20.99 μg ml^−1^) and MCF-7 (IC_50_: 18.89 μg ml^−1^). In addition, the chloroform extract of *S. wallichiana* had remarkable cytotoxic effects on HeLa (IC_50_: 28.53 μg ml^−1^) and HepG-2 (IC_50_: 26.34 μg ml^−1^) cell lines, as reported by Mamadalieva *et al.*^78^ In another study by Mahmoud *et al.*,^79^ the *n*-hexane fraction of *S. succulenta* displayed a good cytotoxic effect on MCF-7 and A549 cell lines with IC_50_ values of 15.5 and 22.6 μg ml^−1^, respectively. Naghibi *et al.*^80^ tested six *Silene* species on several cell lines (A-549, HepG-2, HT-29, MCF-7, and MDBK) and most of them were not active, except for *S. ampullata* on MC-7 (IC_50_: 59.08 μg ml^−1^). The extract of *S. vivianii* exhibited a cytotoxic effect on HepG-2 with an IC_50_ value of 47.96 μg ml^−1^, as reported by Shamkhi *et al.*^81^ When we compared the findings of our study with those in the literature, we found that the ethyl acetate extract of *S. nerimaniae* exhibited significant cytotoxic potential against cell lines, particularly HCT-116. To further interpret this EtOAc-associated HCT-116 cytotoxic profile at the metabolite level, we applied QSAR-based ADEFRank prioritization followed by molecular docking and molecular dynamics analyses.

### HCT-116 QSAR model performance

Two HCT-116-oriented QSAR classification models were constructed from curated ChEMBL bioactivity records to provide activity-evidence layers for ADEFRank-HCT116 prioritization. Their extended performance metrics are summarized in Table S2. For the conservative p60 model, ROC-AUC decreased from 0.9639 in the random split to 0.9352 in the Bemis–Murcko scaffold split, with corresponding MCC values of 0.7933 and 0.7304. The moderate p55 model showed ROC–AUC values of 0.9533 and 0.8996 under random and scaffold splitting, respectively, with MCC values of 0.7512 and 0.5993. The expected reduction in scaffold-split performance indicates a stricter estimate under chemical-series separation, while the retained discrimination supports the use of both models as complementary activity-evidence layers. Accordingly, p60 was used to provide a conservative activity signal, whereas p55 was used to capture a broader moderate-activity signal. These QSAR outputs were interpreted as computational activity-evidence scores and were not considered substitutes for experimentally measured HCT-116 IC50, *K*_i_, or compound-level cytotoxicity data.

### ADEFRank-HCT116 prioritization of *Silene* metabolites

Because candidate rankings were summarized across multiple abundance/QSAR weighting scenarios rather than a single manually selected weight, the hierarchy described below reflects rank-stable, reliability-gated robustness rather than a single deterministic score. The 52 LC–MS-annotated metabolites of *S. nerimaniae* were prioritized using the ADEFRank-HCT116 workflow, with 37 metabolites assigned to the structurally reliable core set and 15 retained as sensitivity-only candidates because of residual structural, positional, or isomeric uncertainty. The core/sensitivity analysis-set assignment is summarized in Table S4. The complete ADEFRank-HCT116 output is provided in Table S3, and the ranking statistics of the prioritized core candidates selected for structure-based follow-up are summarized in Table 8.

**Table 8:** ADEFRank-HCT116-prioritized core candidates selected for primary structure-based follow-up

Candidate	EtOAc (%)	p55 score	p60 score	Reliability gate	Median rank	Top-5 freq.	Pareto front	Role
Diosmetin	17.111	0.566	0.434	1.000	1	1.000	1	Primary robust balanced
Luteolin	35.851	0.090	0.067	1.000	6	0.308	1	Primary abundance-driven
Eriodyctiol	5.873	0.170	0.126	1.000	3	0.744	2	Secondary robust
Acacetin	8.812	0.154	0.097	1.000	5	0.641	2	Secondary robust
Isorhamnetin	0.187	0.311	0.240	1.000	2	0.897	2	Rank-stable, QSAR-supported low-abundance

Within the core set, diosmetin emerged as the primary robust balanced candidate, reflecting combined support from EtOAc abundance, HCT-116-oriented QSAR evidence, full reliability gating, Pareto-front support, and stable top ranking across the scanned weighting scenarios. Luteolin represented the primary abundance-driven candidate because of its dominant EtOAc contribution, although its QSAR-derived activity evidence was weaker than that of diosmetin. Eriodyctiol and acacetin formed a secondary robust group with meaningful EtOAc abundance and consistent cross-scenario support. Isorhamnetin was retained as a rank-stable, QSAR-supported low-abundance observation because it showed strong rank stability despite its low EtOAc contribution.

Diosmin was retained in the complete ADEFRank-HCT116 output as a QSAR-supported low-abundance observation, but it was not included in the primary docking ligand set because of its very low EtOAc abundance, borderline applicability-domain status, reduced reliability gate, and weaker external contextual support relative to the aglycone candidates. Sensitivity-only metabolites, including hydroxy fatty acid derivatives and structurally ambiguous flavonoid conjugates, were reported separately and were not merged into the primary core hierarchy. Accordingly, five core candidates were selected for structure-based follow-up: diosmetin, luteolin, eriodyctiol, acacetin, and isorhamnetin.

### Comparison with abundance-only ranking

To determine whether ADEFRank-HCT116 provided information beyond simple metabolite abundance, the integrated ranking was compared with an abundance-only baseline derived from EtOAc relative abundance among the core candidates (Table S5). This comparison showed that the ADEFRank hierarchy was not a direct reflection of metabolite abundance alone. Luteolin remained the primary abundance-driven candidate, consistent with its dominant EtOAc contribution, whereas diosmetin was prioritized as the primary robust balanced candidate by combining meaningful abundance, stronger HCT-116-oriented QSAR evidence, full reliability gating, and stable cross-scenario ranking.

The most notable rank-shift pattern was observed for isorhamnetin, which moved upward relative to its abundance-only position because of QSAR-supported and rank-stable evidence across the weighting scenarios. However, because its EtOAc abundance was low, isorhamnetin was interpreted as a rank-stable low-abundance observation rather than as a major contributor to the bulk EtOAc extract activity. Eriodyctiol and acacetin showed intermediate profiles, remaining within the secondary robust candidate group through combined abundance and ranking stability rather than being prioritized solely according to abundance. Conversely, Diosmin showed partial rank improvement through QSAR-derived evidence, but its very low EtOAc abundance, borderline applicability-domain status, and reduced reliability gate limited its interpretation to a QSAR-supported low-abundance observation.

Overall, the abundance-only comparison indicates that ADEFRank-HCT116 incorporated QSAR-derived activity evidence, applicability-domain confidence, structure-assignment confidence, and cross-scenario rank stability beyond what abundance alone would suggest. The resulting hierarchy should therefore be interpreted as a reliability-gated, hypothesis-generating prioritization framework rather than as a direct measure of metabolite contribution or experimentally confirmed activity.

### Independent external evidence audit of prioritized candidates

The external evidence audit was used only as post-ranking interpretive support and did not alter the ADEFRank-HCT116 hierarchy (Table S6). No prioritized focus candidate showed direct active HCT-116 viability/cytotoxicity evidence in the screened public resources. ChEMBL auditing further distinguished non-training candidate-level evidence from records overlapping with the QSAR training set; training-overlap records were not treated as independent external support. PubChem BioAssay strict curation provided target-level mechanistic support for diosmetin, luteolin, eriodyctiol, and acacetin, whereas isorhamnetin showed the strongest HCT116 cell-context mechanistic evidence through the IP6K2/InsP7 pathway. Diosmin showed only weak or negative contextual support, and the structurally ambiguous sensitivity-only candidates lacked direct public validation. Therefore, external evidence was interpreted as contextual mechanistic support rather than proof of compound-level HCT-116 cytotoxicity.

### Molecular docking screening of ADEFRank-prioritized candidates

The five primary ADEFRank-HCT116 core candidates were subjected to molecular docking screening against four HCT-116-associated protein targets: PI3Kα, AKT1, GSK-3β, and Bcl-2. The complete 5 × 4 docking matrix is provided in Table S8 and visualized as a binding-energy heatmap in Fig. 3A. Representative docking results used for downstream structure-based interpretation are summarized in Table 9, while docking grid parameters and native ligand-based pocket checks are provided in Table S7. Additional docking pose annotation and MD-selection rationale are provided in Table S9.

**Fig. 3:**
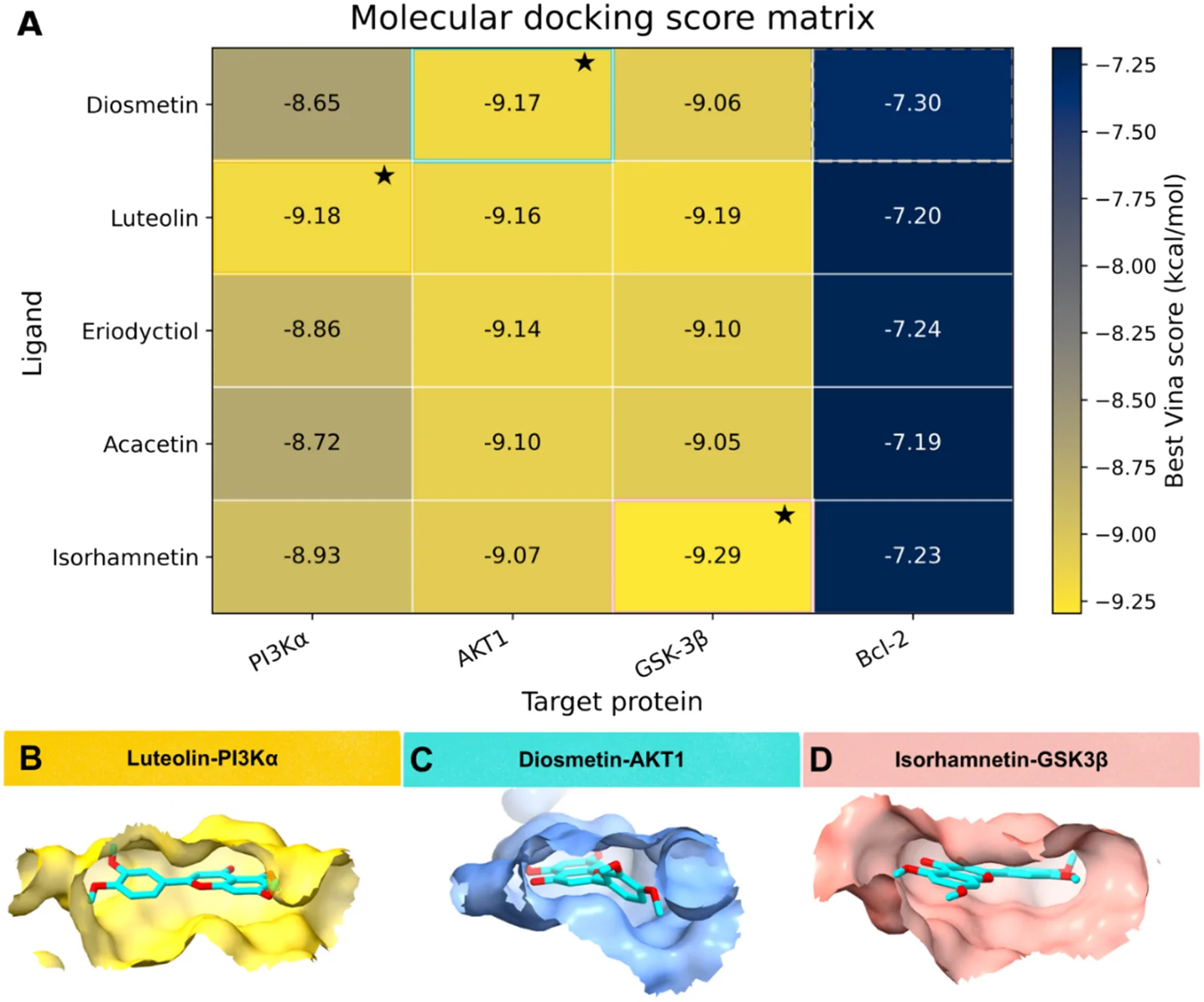
Molecular docking screening of ADEFRank-HCT116-prioritized candidates. (A) Heatmap showing the best AutoDock Vina scores of five prioritized metabolites against four HCT-116-associated protein targets. More negative values indicate more favorable predicted docking scores. Star-marked and color-outlined cells indicate the three representative complexes selected for molecular dynamics simulations: luteolin–PI3Kα, diosmetin–AKT1, and isorhamnetin–GSK-3β. The dashed outline indicates a secondary docking observation for diosmetin–Bcl-2. (B–D) representative binding poses of the selected complexes within the corresponding protein binding pockets: (B) luteolin–PI3Kα, (C) diosmetin–AKT1, and (D) isorhamnetin–GSK-3β.

**Table 9:** Representative docking results selected for molecular dynamics simulations

Complex	PDB ID	Best Vina (kcal mol^−1^)	Est. *K*_i_ (nM)	Pocket proximity (Å)	H-bond cand.	Contacts	Selection status
Luteolin–PI3Kα	4YKN	−9.183	185.700	2.940	7	18	Selected for MD
Diosmetin–AKT1	3CQU	−9.172	189.180	1.118	6	17	Selected for MD
Isorhamnetin–GSK-3β	1Q41	−9.295	153.715	1.707	6	15	Selected for MD

Across the docking screen, the prioritized flavonoid candidates produced consistently favorable predicted Vina score profiles within the investigated target panel. Luteolin showed the best PI3Kα docking profile, with a best Vina score of −9.183 kcal mol^−1^ and a docking-derived estimated *K*_i_ of 185.700 nM, and its representative pose was located within the intended native-ligand-defined binding region (Fig. 3B). Diosmetin showed the leading AKT1 profile, with a best Vina score of −9.172 kcal mol^−1^ and an estimated *K*_i_ of 189.180 nM, together with close native-pocket proximity and an interpretable interaction profile (Fig. 3C). Isorhamnetin produced the strongest GSK-3β docking profile, with a best Vina score of −9.295 kcal mol^−1^ and an estimated *K*_i_ of 153.715 nM, and occupied the intended binding pocket (Fig. 3D).

Representative complexes were selected for molecular dynamics simulations based on target-level docking rank, binding-site occupancy, interaction-profile interpretability, target relevance, and consistency with ADEFRank candidate roles. Accordingly, luteolin–PI3Kα, diosmetin–AKT1, and isorhamnetin–GSK-3β were selected for 100 ns MD simulations (Tables 9, S9 and Fig. 3B–D). Diosmetin–Bcl-2 was retained as a secondary docking observation, whereas luteolin–GSK-3β was considered a backup GSK-3β observation but was not selected as the primary MD representative. These docking results were interpreted as structure-based, hypothesis-generating support for representative complex selection and were not treated as experimentally measured binding affinities or direct target-validation evidence.

### Molecular dynamics simulation analysis of representative complexes

To examine the dynamic behavior of the representative docking-derived complexes, 100 ns MD simulations were performed for luteolin–PI3Kα, diosmetin–AKT1, and isorhamnetin–GSK-3β. Protein backbone RMSD, ligand RMSD, radius of gyration, minimum protein–ligand distance, hydrogen-bond profiles, and protein–ligand contact dynamics are shown in Fig. 4, while RMSF and SASA profiles are provided separately in Fig. S1. Summary trajectory metrics are presented in Table 10, and the full MD metric summary is provided in Table S10.

**Fig. 4:**
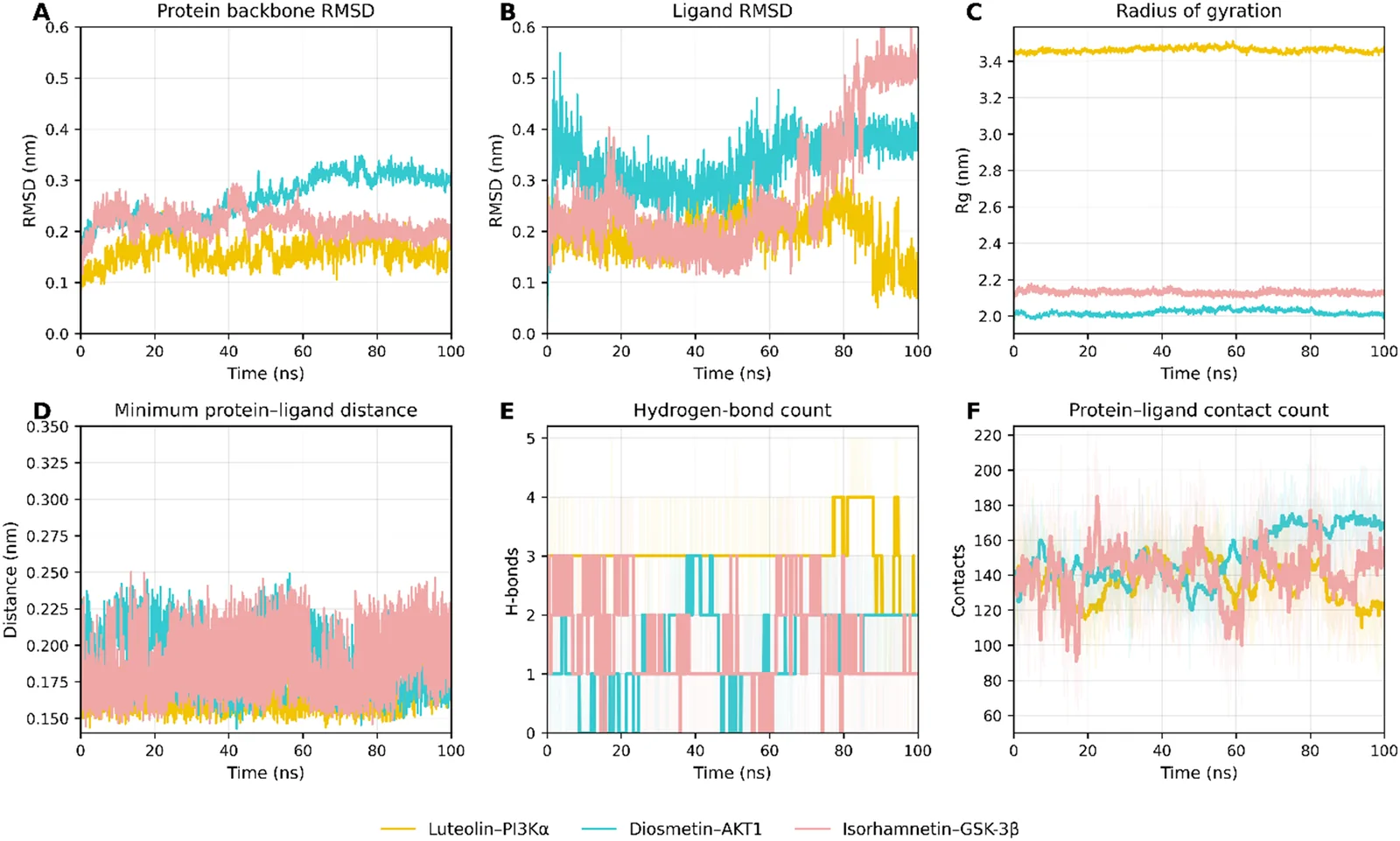
Molecular dynamics stability and interaction-persistence profiles of representative protein–ligand complexes. (A) Protein backbone RMSD, (B) ligand RMSD, (C) radius of gyration, (D) minimum protein–ligand distance, (E) hydrogen-bond count, and (F) protein–ligand contact count over 100 ns simulations for the three representative complexes: luteolin–PI3Kα, diosmetin–AKT1, and isorhamnetin–GSK-3β. Hydrogen-bond and contact-count profiles are shown with smoothed trend lines to emphasize interaction persistence over the trajectory.

**Table 10:** Summary of 100 ns molecular dynamics trajectory metrics for representative protein–ligand complexes

Complex	Protein RMSD mean/max (nm)	Ligand RMSD mean/max (nm)	H-bond mean	Min. distance mean (nm)	Contact mean	Overall interpretation
Luteolin–PI3Kα	0.160/0.239	0.194/0.321	3.09	0.164	136.37	Stable complex with low ligand fluctuation and strong H-bond profile
Diosmetin–AKT1	0.265/0.348	0.331/0.550	1.43	0.188	150.81	Stable but more dynamic complex with sustained protein–ligand contacts
Isorhamnetin–GSK-3β	0.213/0.293	0.275/0.637	1.51	0.187	142.04	Stable protein profile with ligand reorientation within the binding pocket

The luteolin–PI3Kα complex showed the most stable overall trajectory profile among the three systems. Protein and ligand RMSD remained low, with mean values of 0.160 nm and 0.194 nm, respectively. The radius of gyration remained within a narrow range, indicating that the protein maintained compactness during the simulation. Luteolin also showed the strongest hydrogen-bond profile among the simulated complexes, with an average of 3.09 hydrogen bonds. The low mean protein–ligand minimum distance of 0.164 nm and the sustained contact profile further supported that luteolin remained accommodated within the PI3Kα binding region without evidence of ligand dissociation during the analyzed trajectory.

The diosmetin–AKT1 complex displayed a stable but more dynamic behavior. The protein RMSD remained within an acceptable range, with a mean value of 0.265 nm, suggesting moderate conformational adaptation rather than large-scale instability. Diosmetin showed a higher ligand RMSD than luteolin, with a mean value of 0.331 nm, indicating greater mobility within the binding region. However, the radius of gyration and SASA profiles did not indicate major structural opening or loss of compactness, and the ligand maintained close protein contact throughout the simulation. Although the average hydrogen-bond count was lower than that of luteolin–PI3Kα, the low mean minimum distance of 0.188 nm and the high contact profile supported a dynamically retained binding mode.

The isorhamnetin–GSK-3β complex showed stable protein behavior with a more pronounced ligand reorientation pattern. Protein RMSD remained limited, with a mean value of 0.213 nm, and the RMSF profile indicated that most of the protein backbone retained low flexibility. Ligand RMSD was higher than that of luteolin and increased during the late phase of the trajectory, consistent with intra-pocket reorientation rather than ligand dissociation. This interpretation was supported by the low mean protein–ligand minimum distance of 0.187 nm, persistent contact profile, and maintained hydrogen-bond interactions. The radius of gyration and SASA profiles also suggested that the GSK-3β structure remained compact and did not undergo major conformational disruption during the simulation.

Overall, all three representative complexes maintained stable protein–ligand contacts over the 100 ns simulations. Luteolin–PI3Kα showed the most stable ligand RMSD and the strongest hydrogen-bond profile, diosmetin–AKT1 represented a more dynamic but contact-rich complex, and isorhamnetin–GSK-3β showed stable protein behavior with ligand reorientation within the binding pocket. These MD results were interpreted as qualitative dynamic support for the docking-derived representative complexes and were not used as quantitative binding free-energy estimates or experimental target confirmation. Because one 100 ns production trajectory was analyzed for each representative complex, future replicate MD simulations and binding free-energy calculations would be useful to further evaluate trajectory reproducibility and relative binding stability.

Taken together, the experimental and computational findings support a coherent hypothesis-generating interpretation of the EtOAc-associated HCT-116 activity of *S. nerimaniae*. The strong cytotoxic profile of the EtOAc extract provided the biological anchor for the ADEFRank-HCT116 workflow, while the integrated ranking indicated that the prioritized hierarchy was not explained by metabolite abundance alone. Instead, candidate prioritization reflected the combined contribution of EtOAc abundance, HCT-116-oriented QSAR evidence, applicability-domain confidence, structure-assignment reliability, and cross-scenario rank stability. This integrative strategy is particularly relevant for complex natural-product extracts, where our results indicate that metabolite abundance, structural reliability, and biological relevance do not necessarily coincide. Similar integrative computational pharmacology and structure-based modeling strategies have also been used to generate mechanistic hypotheses from multi-layer biological and molecular evidence.^12,14,82^ Accordingly, diosmetin, luteolin, eriodyctiol, acacetin, and isorhamnetin should be interpreted as prioritized candidate contributors to the extract-level effect and as sources of mechanistic hypotheses rather than as experimentally confirmed single-compound drivers of the observed activity.

The biological plausibility of the prioritized candidates is further supported by previous colorectal cancer literature on the selected flavonoids. Diosmetin has been reported to exert anti-proliferative and anti-tumorigenic effects against HCT-116 colorectal cancer cells through mitotic disruption and apoptosis induction.^83^ Luteolin has also been shown to suppress proliferation, block cell-cycle progression, and promote apoptosis-related responses in colorectal cancer cells, including HCT-116 and HT-29, partly through modulation of the MAPK pathway.^84^ In addition, isorhamnetin has been reported to inhibit colon cancer cell growth in HCT-116, HT-29, and SW480 cells through the PI3K-Akt–mTOR pathway.^85^ In the present study, docking and MD simulations extended these literature-supported anticancer contexts into structure-based hypotheses involving luteolin–PI3Kα, diosmetin–AKT1, and isorhamnetin–GSK-3β. However, these findings should be considered computationally supported prioritization results rather than direct pharmacological or target-level validation. Future studies should evaluate the isolated prioritized compounds individually and in combination, determine their HCT-116 cytotoxicity and selectivity profiles, and experimentally examine apoptosis-, cell-cycle-, and pathway-related markers to test the proposed mechanistic links.

## Conclusion

To the best of our knowledge, this study is the first to describe the phytochemical profile and biological properties of *S. nerimaniae*. The findings demonstrated that extracts prepared from the aerial parts were particularly rich in phenolic compounds, especially flavonoids. Biological activities varied among the extracts, with the aqueous and acetone extracts showing the strongest antioxidant effects across most assays. In contrast, extracts obtained using organic solvents exhibited significant enzyme inhibitory activities. Notably, all the extracts produced greater inhibition of both isoenzymes than the standard reference compound. Organic extracts showed cytotoxic effects against both normal and cancer cell lines. Among these, the ethyl acetate extract exhibited the most pronounced HCT-116 cytotoxic profile and thus provided the biological basis for the HCT-116-oriented ADEFRank analysis. ADEFRank-HCT116 prioritized diosmetin, luteolin, eriodyctiol, acacetin, and isorhamnetin by integrating EtOAc abundance, QSAR-derived activity evidence, applicability domain confidence, structure assignment reliability, and cross-scenario rank stability. Molecular docking screening of these candidates predicted favorable docking profiles, while 100 ns molecular dynamics simulations provided qualitative dynamic support for the representative luteolin–PI3Kα, diosmetin–AKT1, and isorhamnetin–GSK-3β complexes. These computational findings should be interpreted as metabolite prioritization and support for mechanistic hypotheses rather than as experimental target validation. Overall, these results indicate that *S. nerimaniae* may serve as a valuable natural source of bioactive compounds with potential applications in the pharmaceutical industry. Further investigations focusing on the isolation of active constituents and clarification of their mechanisms of action are warranted.

## Abbreviations

Abbreviations: Full term
A549: Human lung carcinoma (adenocarcinoma) cell line
ABTS: 2,2′-Azino-bis(3-ethylbenzothiazoline-6-sulfonic acid) radical scavenging assay
ACAE: Acarbose equivalents
AChE: Acetylcholinesterase
ADEFRank: Abundance–domain–evidence fusion ranking (workflow)
ADEFRank-HCT116: HCT-116-oriented abundance–domain–evidence fusion ranking workflow
AKT1: Protein kinase B (isoform 1)
AM1-BCC: Austin model 1 – bond charge correction (partial-charge method)
AmberTools: Software suite for the AMBER molecular-simulation force fields
ATCC: American type culture collection
AUC: Area under the curve
BChE: Butyrylcholinesterase
Bcl-2: B-cell lymphoma 2 (protein)
CID: Collision-induced dissociation
CRL-1459: Normal human colon cell line (ATCC designation)
CUPRAC: Cupric ion reducing antioxidant capacity
DMEM-F12: Dulbecco's modified Eagle medium/nutrient mixture F-12
DMSO: Dimethyl sulfoxide
DPPH: 2,2-Diphenyl-1-picrylhydrazyl radical scavenging assay
EDTAE: EDTA (ethylenediaminetetraacetic acid) equivalents
EtOAc: Ethyl acetate
FBS: Fetal bovine serum
FITC: Fluorescein isothiocyanate
FRAP: Ferric reducing antioxidant power assay
GAE: Gallic acid equivalents
GALAE: Galantamine equivalents
GROMACS: Groningen machine for chemical simulations (MD simulation software)
GSK-3β: Glycogen synthase kinase 3 beta
HCA: Hierarchical cluster analysis
hCA I/hCA II: Human carbonic anhydrase isoenzymes I and II
HCT-116: Human colorectal (colon) cancer cell line
HeLa: Cervical adenocarcinoma cell line
HPLC: High-performance liquid chromatography
HRMS: High-resolution mass spectrometry
IC50: Half-maximal inhibitory concentration
IU: International unit
KAE: Kojic acid equivalents
Ki: Inhibition constant
LC–MS: Liquid chromatography–mass spectrometry
LINCS: Linear constraint solver (bond-constraint algorithm)
MD: Molecular dynamics
MMFF94s: Merck molecular force field 94 s
MTT: 3-(4,5-Dimethylthiazol-2-yl)-2,5-diphenyltetrazolium bromide assay
NaCl: Sodium chloride
NCI-60: National Cancer Institute 60 human tumor cell line panel
NPT: Isothermal–isobaric ensemble (constant number of particles, pressure, temperature)
NVT: Canonical ensemble (constant number of particles, volume, temperature)
OD: Optical density
PBD: Phosphomolybdenum assay (total antioxidant capacity)
PBS: Phosphate-buffered saline
PDB: Protein Data Bank
PDBQT: Protein Data Bank, partial charge (Q), atom type (T) format (AutoDock ligand/receptor file format)
PI: Propidium iodide
PI3Kα: Phosphatidylinositol 3-kinase, alpha isoform
PR-AUC: Precision-recall area under the curve
QSAR: Quantitative structure–activity relationship
RCSB: Research Collaboratory for Structural Bioinformatics
RE: Rutin equivalents
RMSD: Root-mean-square deviation
RMSF: Root-mean-square fluctuation
ROC-AUC: Receiver operating characteristic – area under the curve
RPMI-1640: Roswell Park Memorial Institute 1640 (cell culture medium)
SASA: Solvent-accessible surface area
SD: Standard deviation
SI: Selectivity index
SMILES: Simplified molecular input line entry system
TCA: Tricarboxylic acid
TE: Trolox equivalents
TFC: Total flavonoid content
TIP3P: Transferable intermolecular potential with 3 points (water model)
TPC: Total phenolic content
Tyr: Tyrosinase

## Author contributions

Conceptualization, AE, SY, AIU, UG, GA, BHA, GZ; methodology, AE, SY, FN, UG, ND, IK, IY, IG, GZ; software, AE, UG, AIU, GA; validation, GZ; formal analysis, GZ; investigation, AE, SY, AIU, UG, IG, GA, GZ, resources, MA; data curation, MA, GZ, writing – original draft preparation, AE, SY, ML, BHA, GZ; writing – review and editing, AE, SY, UG, IY, IG, ML visualization, ML.; supervision, GZ.; project administration, GZ.; funding acquisition, GZ. All authors have read and agreed to the published version of the manuscript.

## Conflicts of interest

The authors declare no competing interests.

## Supplementary Material

MD-OLF-D6MD00624H-s001

## Data Availability

The data that supports the findings of this study are available on request from the corresponding author. Supplementary information (SI) is available. See DOI: https://doi.org/10.1039/d6md00624h.
