# Supplementary material for: A multi-evidence computational and experimental strategy for Silene nerimaniae: combining phytochemical characterization, bioactivity assessment, and ADEFRank-HCT-116-based metabolite prioritization
Source: RSC Med Chem. 2026 Sep 3. Online ahead of print. doi: 10.1039/d6md00624h (PMC13656220; doi:10.1039/d6md00624h)
Supplement: Supplementary file 1 [file MD-OLF-D6MD00624H-s001.pdf]

Supplementary Information (SI) for RSC Medicinal Chemistry.
This journal is © The Royal Society of Chemistry 2026

Determination of Antioxidant and Enzyme Inhibitory Effects

Antioxidant (DPPH and ABTS radical scavenging, reducing power (CUPRAC and FRAP), phosphomolybdenum and metal chelating (ferrozine method)) and enzyme inhibitory activities (cholinesterase (Elmann’s method), tyrosinase (dopachrome method), α-amylase (iodine/potassium iodide method), α -glucosidase (chromogenic PNPG method) and pancreatic lipase (p-nitrophenyl butyrate (p-NPB) method) were determined.

For the DPPH (1,1-diphenyl-2-picrylhydrazyl) radical scavenging assay: Sample solution was added to 4 mL of a 0.004% methanol solution of DPPH. The sample absorbance was read at 517 nm after a 30 min incubation at room temperature in the dark. DPPH radical scavenging activity was expressed as milligrams of trolox equivalents (mg TE/g extract) [1].

For ABTS (2,2′-azino-bis(3-ethylbenzothiazoline) 6-sulfonic acid) radical scavenging assay: Briefly, ABTS+ was produced directly by reacting 7 mM ABTS solution with 2.45 mM potassium persulfate and allowing the mixture to stand for 12–16 in the dark at room temperature. Prior to beginning the assay, ABTS solution was diluted with methanol to an absorbance of 0.700 ± 0.02 at 734 nm. Sample solution was added to ABTS solution (2 mL) and mixed. The sample absorbance was read at 734 nm after a 30 min incubation at room temperature. The ABTS radical scavenging activity was expressed as milligrams of trolox equivalents (mg TE/g extract) [2].

For CUPRAC (cupric ion reducing activity) activity assay: Sample solution was added to premixed reaction mixture containing CuCl2 (1 mL, 10 mM), neocuproine (1 mL, 7.5 mM) and NH4Ac buffer (1 mL, 1 M, pH 7.0). Similarly, a blank was prepared by adding sample solution (0.5 mL) to premixed reaction mixture (3 mL) without CuCl2 . Then, the sample and blank absorbances were read at 450 nm after a 30 min incubation at room temperature. The absorbance of the blank was subtracted from that of the sample. CUPRAC activity was expressed as milligrams of trolox equivalents (mg TE/g extract) [3].

For FRAP (ferric reducing antioxidant power) activity assay: Sample solution was added to premixed FRAP reagent (2 mL) containing acetate buffer (0.3 M, pH 3.6), 2,4,6-tris(2-pyridyl)-S-triazine (TPTZ) (10 mM) in 40 mM HCl and ferric chloride (20 mM) in a ratio of 10:1:1 (v/v/v). Then, the sample absorbance was read at 593 nm after a 30 min incubation at room temperature. FRAP activity was expressed as milligrams of trolox equivalents (mg TE/g extract) [4].

For phosphomolybdenum method: Sample solution was combined with 3 mL of reagent solution (0.6 M sulfuric acid, 28 mM sodium phosphate and 4 mM ammonium molybdate). The sample absorbance was read at 695 nm after a 90 min incubation at 95 °C. The total antioxidant capacity was expressed as millimoles of trolox equivalents (mmol TE/g extract) [5].

For metal chelating activity assay: Briefly, sample solution was added to FeCl2 solution (0.05 mL, 2 mM). The reaction was initiated by the addition of 5 mM ferrozine (0.2 mL). Similarly, a blank was prepared by adding sample solution (2 mL) to FeCl2 solution (0.05 mL, 2 mM) and water (0.2 mL) without ferrozine. Then, the sample and blank absorbances were read at 562 nm after 10 min incubation at room temperature. The absorbance of the blank was subtracted from that of the sample. The metal chelating activity was expressed as milligrams of EDTA (disodium edetate) equivalents (mg EDTAE/g extract) [6].

For Cholinesterase (ChE) inhibitory activity assay: Sample solution (was mixed with DTNB (5,5-dithio-bis(2-nitrobenzoic) acid, Sigma, St. Louis, MO, USA) (125 µL) and AChE (acetylcholinesterase (Electric ell acetylcholinesterase, Type-VI-S, EC 3.1.1.7,Sigma)), or BChE (butyrylcholinesterase (horse serum butyrylcholinesterase, EC 3.1.1.8, Sigma)) solution (25 µL) in Tris–HCl buffer (pH 8.0) in a 96-well microplate and incubated for 15 min at 25 °C. The reaction was then initiated with the addition of acetylthiocholine iodide (ATCI, Sigma) or butyrylthiocholine chloride (BTCl, Sigma) (25 µL). Similarly, a blank was prepared by adding sample solution to all reaction reagents without enzyme (AChE or BChE) solution. The sample and blank absorbances were read at 405 nm after 10 min incubation at 25 °C. The absorbance of the blank was subtracted from that of the sample and the cholinesterase inhibitory activity was expressed as galanthamine equivalents (mg GALAE/g extract) [7].

For Tyrosinase inhibitory activity assay: Sample solution was mixed with tyrosinase solution (40 µL, Sigma) and phosphate buffer (100 µL, pH 6.8) in a 96-well microplate and incubated for 15 min at 25 °C. The reaction was then initiated with the addition of L-DOPA (40 µL, Sigma). Similarly, a blank was prepared by adding sample solution to all reaction reagents without enzyme (tyrosinase) solution. The sample and blank absorbances were read at 492 nm after a 10 min incubation at 25 °C. The absorbance of the blank was subtracted from that of the sample and the tyrosinase inhibitory activity was expressed as kojic acid equivalents (mgKAE/g extract) [8].

For α-amylase inhibitory activity assay: Sample solution was mixed with α-amylase solution (ex-porcine pancreas, EC 3.2.1.1, Sigma) (50 µL) in phosphate buffer (pH 6.9 with 6 mM sodium chloride) in a 96-well microplate and incubated for 10 min at 37 °C. After pre-incubation, the reaction was initiated with the addition of starch solution (50 µL, 0.05%). Similarly, a blank was prepared by adding sample solution to all reaction reagents without enzyme (α-amylase) solution. The reaction mixture was incubated 10 min at 37 °C. The reaction was then stopped with the addition of HCl (25 µL, 1 M). This was followed by addition of the iodine-potassium iodide solution (100 µL). The sample and blank absorbances were read at 630 nm. The absorbance of the blank was subtracted from that of the sample and the α-amylase inhibitory activity was expressed as acarbose equivalents (mmol ACE/g extract) [9].

Human carbonic anhydrase enzyme was purified by Sepharose 4 B-L-tyrosine sulfanilamide affinity column and characterized by SDS-PAGE as previously applied [10]. The esterase activity of the carbonic anhydrase (CA) enzyme yields a yellow-colored aromatic p-nitrophenolate compound

at a pH of around pH=7.5. By taking advantage of this transformation, the esterase activity of CA enzymes can be determined spectrophotometrically(Tel et al., 2025a)· Various concentrations of the samples in 0.05 M pH=7.4 Tris-SO_4, 0.07 mM of p-nitrophenylacetate (in 1:25 acetone: water), and 20 µL of the enzyme were gently vortexed, and as soon the as the addition of the enzyme, the absorbance change at 348 nm was monitored by measuring minute intervals for three minutes. Control reactions and blank reactions were set up without inhibitors and enzymes. These steps were repeated until more than half of the enzyme activity was inhibited. All data were processed, and graphs were created using GraphPad Prism 8.0.2. IC_{50} value refers in this study to the concentration of an inhibitor needed to inhibit an enzyme activity response by 50%. The calculation of the IC_{50} values was performed using the GraphPad Prism 8.4.0 non-linear regression-[inhibitor]-normalized response (y values 100 down to 0) model.

Table S1. Normalized peak areas obtained from full scan MS spectra.

**Compound name**	***Silene nerimaniae* acetone**	***Silene nerimaniae* acetone water**	***Silene nerimaniae* Ethyl acetate**	***Silene nerimaniae* water**
**Sucrose**	473269703	354068203	32157270	494534787
**Glucose**	64761434	57465841	7544899	300958000
**Arabinose**	11851848	7279825	12288914	85593315
**Galacturonic acid**	14309962	11246421	2869798	728523782
**Malic acid**	17826102	13901597	22187647	4239689860
**Citric acid**	6411550	3814220	4399779	664882819
**Glutaric acid**	2556358	2687424	6017276	22003882
**Vanillic acid hexoside**	1834611	1696487	452339	18006445
**Gallic acid hexoside**	ND	ND	179841	31019504
**Dihydroxybenzoic acid hexoside**	861025	788040	95435	45262320
**Dihydroxybenzoic acid**	33567348	21706648	17008780	50451773
**Hydroxybenzoic acid hexoside**	ND	ND	ND	38498515

**Hydroxybenzoic acid 1**	13113891	16641576	9284126	30076719
**Coumaric acid hexoside**	ND	ND	ND	64002254
**p-Coumaric acid**	ND	5648817	4394930	13047552
**Ferulic acid**	4598872	4438624	3331418	17525285
**Hydroxybenzoic acid 2**	8543760	894848	1506541	26163841
**Apigenin 6-C-hexoside-7-O-hexoside (Saponarin)**	32540856	30400762	1016177	355455733
**Apigenin 6-C-(2"-pentosyl)-hexoside-7-O-hexoside**	77870431	71199301	2998408	1480640591
**Malonyl-apigenin 6-C-(2"-pentosyl)-hexoside-7-O-hexoside**	ND	3175264	2274142	923288966
**Apigenin 6-C-(2"-pentosyl)-hexoside-7-O-(6"-feruloyl)-hexoside**	32620953	32279682	1556172	493663487
**Malonyl-apigenin 6-C-(2"-pentosyl)-hexoside-7-O-(6"-feruloyl)-hexoside**	1162747	2674163	1301508	423988848
**Apigenin 6-C-(2"-pentosyl)-hexoside-7-O-(6"-dihydroferuloyl)-hexoside**	18546416	18618705	720594	288755322
**Luteolin 6-C-hexoside-8-C-pentoside (Carlinoside)**	13202616	14622056	399315	236528844
**Luteolin 6-C-hexoside (Isoorientin)**	22879293	26599470	ND	95629135
**Apigenin 6-C-(2"-pentosyl)-hexoside**	305742438	315471012	25724384	1378715043
**Apigenin 6-C-hexoside (Isovitexin)**	176675011	180758398	38758871	353747311
**Malonyl-apigenin 6-C-(2"-pentosyl)-hexoside**	1180185	2345190	516366	278460591
**Chrysoeriol 6-C-hexoside (Isoscoparin)**	ND	4821191	ND	23691235
**Quercetin 3-O-(6"-rhamnosyl)-hexoside (Rutin)**	67744853	78497728	2738986	209708988
**Quercetin 3-O-hexoside (Hyperoside)**	31139738	33867524	7236232	71413424
**Kaempferol 3-O-(6"-rhamnosyl)-hexoside (Nicotiflorin)**	46965826	51050058	4657992	196282078
**Kaempferol 3-O-hexoside (Astragalin)**	14300509	15501798	6727433	34328445
**Apigenin 7-O-(6"-rhamnosyl)-hexoside (Isorhoifolin)**	26829809	26497707	6005509	94101571
**Apigenin 7-O-hexoside (Apigenitrin)**	14241563	13614163	7644201	13131042
**Diosmetin 7-O-(6"-rhamnosyl)-hexoside (Diosmin)**	4456942	3892418	1293335	8285065
**Luteolin 7-O-hexoside (Cynaroside)**	7947025	7877132	4039368	5296898
**Rhamnetin 3-O-hexoside**	16100844	14811932	9219920	9022664
**Taxifolin**	14240672	16972736	13224001	ND
**Luteolin**	6215327243	5712171348	4894636369	921693380
**Eriodyctiol**	1018082564	942082948	801879803	147456851

**Isorhamnetin**	26040578	26228114	25513835	3016001
**Naringenin**	156021477	151900125	149461071	17496206
**Kaempferol**	256495624	215994072	298574421	ND
**Diosmetin**	2890642304	2549612214	2336161607	454001604
**Rhamnetin**	51624260	57604923	45692968	7371609
**Acacetin**	1323398034	1206721609	1203143097	120765343
**Trihydroxyoctadecadienoic acid**	284322403	255258417	158958744	858863188
**Hydroxyoctadecatrienoic acid**	672964111	533818345	261826402	228727968
**Hydroxyoctadecadienoic acid**	604352504	545039081	773925431	79669653
**Hydroxyoctadecanoic acid**	1216972477	935058537	778269461	232968720
**Hydroxyeicosanoic acid**	2180965734	1755182226	1663028508	270990731

ND – not detected.

Table S2. Performance of HCT-116-oriented QSAR models used as ADEFRank activity-evidence layers.

**Model**	**Cutoff**	**Split**	**nA**	**nI**	**TestA**	**TestI**	**Acc**	**BA**	**ROC**	**PR**	**F1**	**MCC**	**Sen**	**Spe**	**TN**	**FP**	**FN**	**TP**
**p60**	A ≥ 6.0; I < 5.0	Random	8050	6092	1610	1219	0.8978	0.8984	0.9639	0.9716	0.9088	0.7933	0.8944	0.9024	1100	119	170	1440
**p60**	A ≥ 6.0; I < 5.0	BM scaffold	8050	6092	1627	1234	0.8637	0.8685	0.9352	0.9497	0.8743	0.7304	0.8334	0.9036	1115	119	271	1356
**p55**	A ≥ 5.5; I < 5.0	Random	11961	6092	2392	1219	0.8898	0.8711	0.9533	0.9748	0.9178	0.7512	0.9285	0.8138	992	227	171	2221
**p55**	A ≥ 5.5; I < 5.0	BM scaffold	11961	6092	2347	1264	0.8228	0.7757	0.8996	0.9430	0.8725	0.5993	0.9327	0.6187	782	482	158	2189

Table Note: A, active; I, inactive; BM, Bemis–Murcko; Acc, accuracy; BA, balanced accuracy; PR, PR-AUC/average precision; Sen, sensitivity; Spe, specificity. Sensitivity and specificity were calculated from the confusion matrix using a probability threshold of 0.50. Gray-zone records were excluded from the corresponding model training. QSAR outputs were used only as computational activity-evidence layers within ADEFRank, not as direct experimental HCT-116 activity values.

Table S3. Complete ADEFRank-HCT116 output for all LC-MS-annotated metabolites of *Silene nerimaniae*.

Compound	Analysis set	EtOAc (%)	p55 score	p60 score	Reliability gate	Median rank	Top-5 freq.	Pareto front	Priority class
**Diosmetin**	Core	17.111	0.566	0.434	1.000	1	1.000	1	Primary robust balanced
**Luteolin**	Core	35.851	0.090	0.067	1.000	6	0.308	1	Primary abundance-driven
**Eriodyctiol**	Core	5.873	0.170	0.126	1.000	3	0.744	2	Secondary robust
**Acacetin**	Core	8.812	0.154	0.097	1.000	5	0.641	2	Secondary robust
**Isorhamnetin**	Core	0.187	0.311	0.240	1.000	2	0.897	2	Rank-stable QSAR-low
**Diosmetin 7-O-(6"-rhamnosyl)-hexoside (Diosmin)**	Core	0.009	0.486	0.388	0.837	9	0.385	2	QSAR-supported low-abund.
**Malonyl-apigenin 6-C-(2"-pentosyl)-hexoside-7-O-(6"-feruloyl)-hexoside**	Core	0.010	0.463	0.263	0.771	17	0.179	2	QSAR-supported low-abund.
**Malonyl-apigenin 6-C-(2"-pentosyl)-hexoside-7-O-hexoside**	Core	0.017	0.398	0.287	0.771	14	0.000	2	QSAR-supported low-abund.
**Sucrose**	Core	0.236	0.223	0.159	0.837	10	0.000	2	Low-priority small metabolite
**Glucose**	Core	0.055	0.140	0.114	0.837	27	0.000	4	Low-priority small metabolite
**Arabinose**	Core	0.090	0.307	0.201	0.592	23	0.000	4	Low-priority small metabolite
**Galacturonic acid**	Core	0.021	0.261	0.162	0.592	32	0.000	5	Low-priority small metabolite
**Malic acid**	Core	0.163	0.321	0.210	0.592	19	0.000	3	Low-priority small metabolite
**Citric acid**	Core	0.032	0.237	0.096	0.592	32	0.000	5	Low-priority small metabolite
**Glutaric acid**	Core	0.044	0.140	0.088	0.592	35	0.000	5	Low-priority small metabolite
**p-Coumaric acid**	Core	0.032	0.151	0.126	1.000	23	0.000	4	Lower-priority core
**Ferulic acid**	Core	0.024	0.099	0.093	1.000	32	0.000	6	Lower-priority core
**Apigenin 6-C-hexoside-7-O-hexoside (Saponarin)**	Core	0.007	0.130	0.105	1.000	35	0.000	6	Lower-priority core
**Apigenin 6-C-(2"-pentosyl)-hexoside-7-O-hexoside**	Core	0.022	0.335	0.246	0.771	19	0.000	2	Lower-priority core
**Apigenin 6-C-(2"-pentosyl)-hexoside-7-O-(6"-feruloyl)-hexoside**	Core	0.011	0.394	0.249	0.771	19	0.000	3	Lower-priority core
**Apigenin 6-C-(2"-pentosyl)-hexoside-7-O-(6"-**	Core	0.005	0.394	0.275	0.771	24	0.000	3	Lower-priority core

**dihydroferuloyl)-hexoside**									
**Luteolin 6-C-hexoside-8-C-pentoside (Carlinoside)**	Core	0.003	0.338	0.276	1.000	14	0.256	2	Lower-priority core
**Luteolin 6-C-hexoside (Isoorientin)**	Core	0.000	0.131	0.125	1.000	37	0.000	6	Lower-priority core
**Apigenin 6-C-hexoside (Isovitexin)**	Core	0.284	0.071	0.079	1.000	20	0.000	4	Lower-priority core
**Malonyl-apigenin 6-C-(2"-pentosyl)-hexoside**	Core	0.004	0.488	0.351	0.495	31	0.000	3	Lower-priority core
**Chrysoeriol 6-C-hexoside (Isoscoparin)**	Core	0.000	0.274	0.267	1.000	27	0.051	3	Lower-priority core
**Quercetin 3-O-(6"-rhamnosyl)-hexoside (Rutin)**	Core	0.020	0.247	0.195	1.000	15	0.000	3	Lower-priority core
**Quercetin 3-O-hexoside (Hyperoside)**	Core	0.053	0.140	0.108	1.000	22	0.000	4	Lower-priority core
**Kaempferol 3-O-(6"-rhamnosyl)-hexoside (Nicotiflorin)**	Core	0.034	0.226	0.191	1.000	12	0.000	3	Lower-priority core
**Kaempferol 3-O-hexoside (Astragalin)**	Core	0.049	0.117	0.097	1.000	27	0.000	5	Lower-priority core
**Apigenin 7-O-(6"-rhamnosyl)-hexoside (Isorhoifolin)**	Core	0.044	0.331	0.152	0.837	15	0.000	3	Lower-priority core
**Apigenin 7-O-hexoside (Apigenitrin)**	Core	0.056	0.180	0.068	0.922	23	0.000	4	Lower-priority core
**Luteolin 7-O-hexoside (Cynaroside)**	Core	0.030	0.207	0.092	1.000	22	0.000	5	Lower-priority core
**Taxifolin**	Core	0.097	0.178	0.122	1.000	10	0.000	3	Lower-priority core
**Naringenin**	Core	1.095	0.111	0.080	1.000	11	0.000	3	Lower-priority core
**Kaempferol**	Core	2.187	0.060	0.060	1.000	15	0.000	3	Lower-priority core
**Rhamnetin**	Core	0.335	0.190	0.157	1.000	5	0.538	2	Lower-priority core
**Hydroxyeicosanoic acid**	Sensitivity-only	12.181	0.481	0.361	0.620	1	1.000	1	Sensitivity high-abund.
**Hydroxyoctadecanoic acid**	Sensitivity-only	5.700	0.377	0.222	0.620	3	1.000	2	Sensitivity high-abund.
**Hydroxyoctadecadienoic acid**	Sensitivity-only	5.669	0.299	0.199	0.620	4	0.897	3	Sensitivity high-abund.
**Apigenin 6-C-(2"-pentosyl)-hexoside**	Sensitivity-only	0.188	0.307	0.258	0.742	2	1.000	1	Sensitivity robust low-abund.
**Vanillic acid hexoside**	Sensitivity-only	0.003	0.366	0.336	0.620	10	0.128	2	Lower-priority sensitivity
**Gallic acid hexoside**	Sensitivity-only	0.001	0.262	0.129	0.696	11	0.000	2	Lower-priority sensitivity
**Dihydroxybenzoic acid hexoside**	Sensitivity-only	0.001	0.353	0.262	0.742	9	0.256	1	Lower-priority sensitivity
**Dihydroxybenzoic acid**	Sensitivity-only	0.125	0.166	0.116	0.742	8	0.256	2	Lower-priority sensitivity
**Hydroxybenzoic acid hexoside**	Sensitivity-only	0.000	0.276	0.127	0.620	13	0.000	4	Lower-priority sensitivity
**Hydroxybenzoic acid 1**	Sensitivity-	0.068	0.079	0.051	0.620	12	0.000	4	Lower-priority

	only								sensitivity
**Coumaric acid hexoside**	Sensitivity-only	0.000	0.193	0.103	0.620	14	0.000	5	Lower-priority sensitivity
**Hydroxybenzoic acid 2**	Sensitivity-only	0.011	0.104	0.064	0.620	15	0.000	4	Lower-priority sensitivity
**Rhamnetin 3-O-hexoside**	Sensitivity-only	0.068	0.216	0.165	0.742	6	0.333	2	Lower-priority sensitivity
**Trihydroxyoctadecadienoic acid**	Sensitivity-only	1.164	0.454	0.314	0.439	7	0.103	2	Sensitivity QSAR-supported
**Hydroxyoctadecatrienoic acid**	Sensitivity-only	1.918	0.426	0.297	0.439	7	0.026	2	Sensitivity QSAR-supported

Table note: p55 and p60 scores represent HCT-116-oriented QSAR-derived activity-evidence scores and should not be interpreted as experimental activity values. Reliability gate summarizes the geometric mean of applicability-domain confidence and structure-assignment confidence. Median rank, top-5 frequency, and Pareto-front assignment summarize the reliability-gated multi-scenario ADEFRank-HCT116 prioritization. EtOAc (%) refers to the relative abundance contribution in the ethyl acetate extract. Priority class indicates the final qualitative interpretation assigned after multi-scenario ranking; “QSAR-low” and “low-abund.” indicate QSAR-supported low-abundance candidates.

Table S4. Structure-curation status and ADEFRank analysis-set assignment of LC-MS-annotated metabolites.

Analysis set	No. of metabolites	Structure-curation status	Use in ADEFRank-HCT116	Downstream interpretation
**Core**	37	Metabolites with sufficiently resolved structures and valid molecular representations	Included in the primary reliability-gated ADEFRank-HCT116 ranking	Used to define the primary prioritization hierarchy and ligand candidates for structure-based follow-up
**Sensitivity-only**	15	Metabolites with residual positional, isomeric, or structural ambiguity despite LC-MS annotation	Retained in a separate sensitivity analysis rather than merged into the primary core hierarchy	Reported as chemically relevant observations but not used to define the primary docking ligand set

The analysis-set assignment was based on structure-curation confidence after LC-MS annotation. Core metabolites were considered suitable for primary ADEFRank-HCT116 prioritization because their structures could be represented with sufficient confidence for QSAR and reliability-gated ranking. Sensitivity-only metabolites were retained to preserve chemically relevant LC-MS annotations but were not merged into the primary core hierarchy because of residual structural, positional, or isomeric uncertainty.

Table S5. Comparison of abundance-only ranking and ADEFRank-HCT116 integrated ranking among core candidates.

Candidate	EtOAc (%)	Abundance-only rank	ADEFRank median rank	Rank shift	Interpretation
**Luteolin**	35.851	1	6	Moderated	Highest-abundance metabolite; retained as primary abundance-driven candidate despite weaker QSAR support
**Diosmetin**	17.111	2	1	Upgraded	Prioritized as primary robust balanced candidate through combined abundance, QSAR support, and reliability
**Acacetin**	8.812	3	5	Slightly moderated	High-abundance flavonoid retained within the secondary robust candidate group
**Eriodyctiol**	5.873	4	3	Upgraded	Moderate-abundance flavonoid upgraded through cross-scenario rank stability
**Isorhamnetin**	0.187	10	2	Rank-stability upgrade	Low-abundance metabolite upgraded through QSAR-supported rank stability; interpreted as a low-abundance observation rather than a major extract contributor
**Diosmin**	0.009	31	9	Partially upgraded	Very low-abundance QSAR-supported observation; retained in the complete ADEFRank output but excluded from the primary docking ligand set due to reduced reliability and lower integrated priority

Abundance-only rank was calculated among the 37 structurally reliable core metabolites using EtOAc relative abundance in descending order. ADEFRank median rank summarizes the reliability-gated multi-scenario integrated ranking. Lower rank values indicate higher priority. Rank shift describes the direction of change from abundance-only ranking to ADEFRank-HCT116 integrated ranking.

Table S6. Summary of independent external evidence audit for ADEFRank-HCT116 focus candidates.

Evidence class	Candidate(s) or group	Direct HCT-116 viability/cytotoxicity support	Interpretation
**HCT116 cell-context mechanistic support**	Isorhamnetin	ND	IP6K2/InsP7-related HCT116 mechanistic evidence; not direct viability validation
**Target-level mechanistic support**	Diosmetin, Luteolin, Eriodyctiol, Acacetin	ND	Mechanistic plausibility support; not direct HCT-116 cytotoxicity validation
**Weak or negative contextual support**	Diosmin	ND	Retained only as a QSAR-supported low-abundance observation
**No usable external evidence**	Sensitivity-only and structurally ambiguous candidates	ND	Reported separately; absence of public evidence does not imply inactivity

Table note: External evidence was used only for post-ranking interpretation and did not modify ADEFRank-HCT116 scores. ND, not detected under the applied audit criteria. ChEMBL training-overlap and connectivity-only matches were not treated as independent validation.

Table S7. Native ligand-based docking-site validation and pocket checks for selected protein targets.

Target	PDB ID	Native ligand	Grid center X, Y, Z (Å)	Grid size X, Y, Z (Å)	Check type	Result (Å)	Interpretation
**PI3Kα**	4YKN	4EL	−0.068, 2.733, −39.140	26.000, 28.000, 24.000	Local-pose geometry check	0.6024	Acceptable local-pose geometry; global redocking was not used as the primary validation metric
**AKT1**	3CQU	CQU	4.839, 3.602, 17.854	24.000, 24.000, 24.000	Global redocking RMSD	1.0219	Acceptable redocking validation
**GSK-3β**	1Q41	IXM	39.632, 6.297, 36.269	24.000, 26.000, 24.000	Global redocking RMSD	0.8729	Acceptable redocking validation
**Bcl-2**	6O0K	LBM	−15.323, 2.214, −9.593	30.000, 30.000, 36.000	Rigid native-ligand local-pose RMSD	0.1949	Acceptable local-pose validation with rigid-native note

Table Note: Docking grids were defined according to the corresponding co-crystallized ligand-binding regions. Grid center and grid size values are reported in Å. Native ligand-based checks were performed before production docking. RMSD/local-pose values are reported in Å. For PI3Kα, the local-pose geometry check was used because the global redocking result was not interpreted as a classical successful redocking validation. Native-to-docked centroid distances of Silene metabolites were used separately as binding-site proximity indicators and were not interpreted as redocking validation metrics.

Table S8. Complete 5 × 4 molecular docking matrix of ADEFRank-HCT116-prioritized candidates against selected HCT-116-associated targets.

Ligand	PI3Kα / 4YKN	AKT1 / 3CQU	GSK-3β / 1Q41	Bcl-2 / 6O0K
**Diosmetin**	−8.651 (−8.631 ± 0.016)	**−9.172 (−9.161 ± 0.009)**	−9.064 (−9.049 ± 0.011)	−7.303 (−7.297 ± 0.006)†
**Luteolin**	**−9.183 (−9.150 ± 0.023)**	−9.160 (−9.145 ± 0.008)	−9.189 (−9.183 ± 0.007) ‡	−7.197 (−7.178 ± 0.013)
**Eriodyctiol**	−8.862 (−8.842 ± 0.018)	−9.143 (−9.123 ± 0.011)	−9.096 (−9.078 ± 0.008)	−7.237 (−7.223 ± 0.013)
**Acacetin**	−8.722 (−8.679 ± 0.024)	−9.100 (−9.081 ± 0.012)	−9.053 (−9.039 ± 0.012)	−7.188 (−7.179 ± 0.006)
**Isorhamnetin**	−8.927 (−8.851 ± 0.028)	−9.072 (−9.039 ± 0.023)	**−9.295 (−9.273 ± 0.011)**	−7.226 (−7.220 ± 0.004)

Table Note: Values are reported as best Vina score followed by mean ± SD in parentheses, calculated from 10 independent docking runs for each ligand–target pair. All values are given in kcal/mol. Bold entries indicate complexes selected for molecular dynamics simulations. †Secondary docking observation. ‡Backup GSK-3β docking observation.

Table S9. Docking pose annotation and MD-selection rationale for representative ligand–target complexes.

Complex	Best Vina (kcal/mol)	Pocket proximity (Å)	H-bond cand.	Contacts	Docking interpretation	Status
**Luteolin–PI3Kα**	−9.183	2.940	7	18	Best PI3Kα profile with native-pocket proximity support	Selected for MD
**Diosmetin–AKT1**	−9.172	1.118	6	17	Best AKT1 profile with close native-pocket proximity	Selected for MD
**Isorhamnetin–GSK-3β**	−9.295	1.707	6	15	Best GSK-3β profile and strongest overall docking score	Selected for MD
**Diosmetin–Bcl-2**	−7.303	7.253	4	11	Best Bcl-2 score but near-pocket/review-level observation	Secondary
**Luteolin–GSK-3β**	−9.189	1.844	5	14	Strong GSK-3β profile but not selected because Isorhamnetin–GSK-3β was the primary representative	Backup

Pocket proximity refers to the native-ligand-defined binding-site proximity indicator and was not interpreted as a redocking RMSD metric. H-bond cand., hydrogen-bond candidates identified by geometry-based contact analysis. Contacts indicate the number of nearby protein–ligand contact residues/features detected for the representative docking pose. MD selection was based on docking rank, binding-site occupancy, interaction-profile interpretability, target relevance, and consistency with ADEFRank candidate roles.

Table S10. Full molecular dynamics summary metrics for representative protein–ligand complexes.

Complex	Prot. RMSD	Lig. RMSD	RMSF	Rg	SASA	H-bond	Min. dist.	Contacts
**Luteolin–PI3Kα**	0.160 / 0.239	0.194 / 0.321	0.171 / 0.582	3.463	562.37	3.09	0.164	136.37
**Diosmetin–AKT1**	0.265 / 0.348	0.331 / 0.550	0.157 / 0.872	2.020	165.78	1.43	0.188	150.81
**Isorhamnetin–GSK-3β**	0.213 / 0.293	0.275 / 0.637	0.120 / 0.819	2.130	179.17	1.51	0.187	142.04

Values for Prot. RMSD, Lig. RMSD, and RMSF are reported as mean / max in nm. Rg and Min. dist. are reported as mean values in nm; SASA is reported as mean value in nm^2. H-bond and Contacts indicate mean protein–ligand interaction counts over the 100 ns trajectories. These metrics describe trajectory stability and interaction persistence, not quantitative binding free-energy estimates.

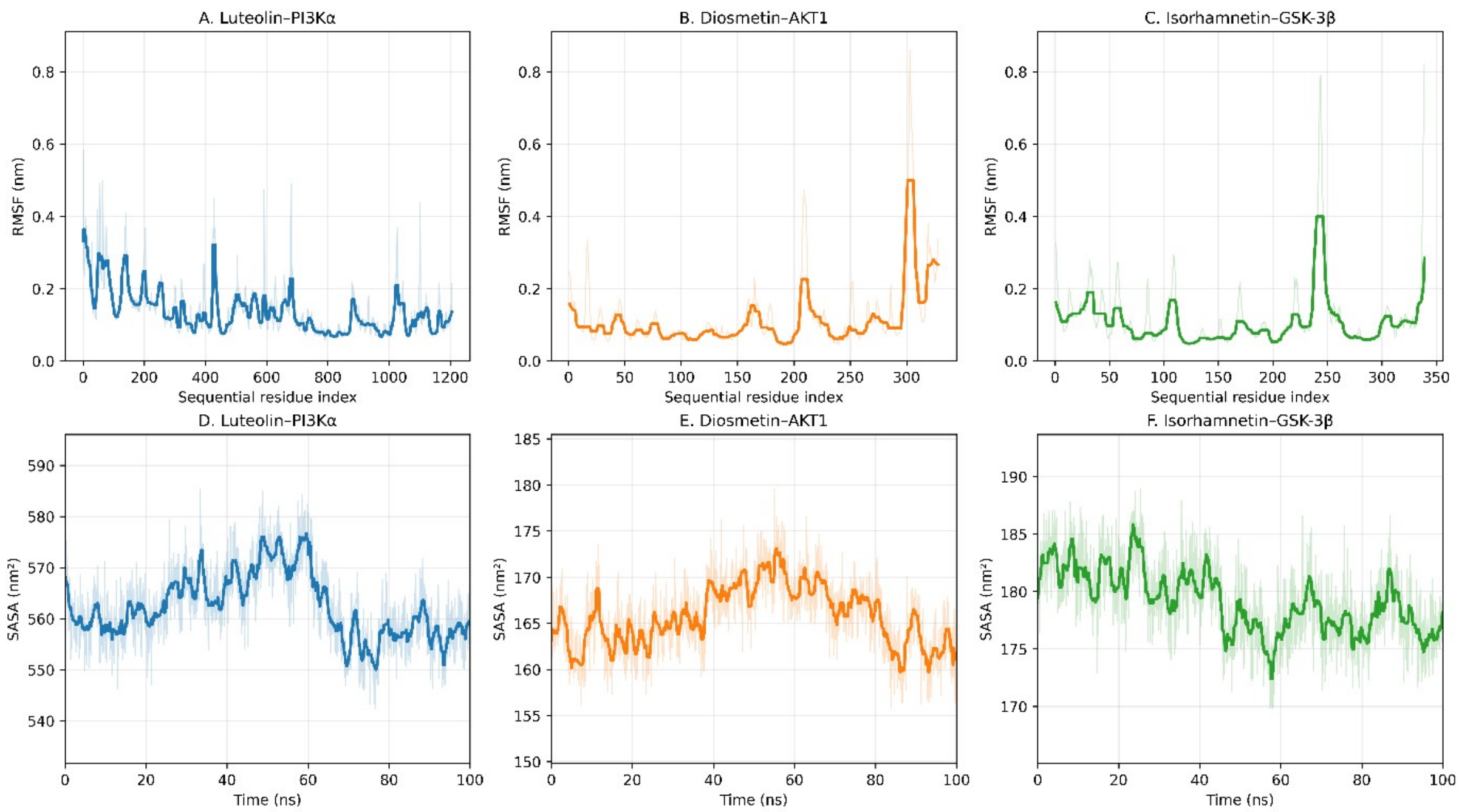

Figure S1. RMSF and SASA profiles of representative protein–ligand complexes. Panels A–C show protein Cα RMSF profiles for Luteolin–PI3Kα, Diosmetin–AKT1, and Isorhamnetin–GSK-3β, respectively, plotted according to sequential residue index rather than original residue numbering because residue numbering and chain organization differ among the analyzed protein structures. Panels D–F show the corresponding solvent-accessible surface area (SASA) profiles over the 100 ns simulations. Faint lines indicate raw values, and solid lines indicate rolling-median-smoothed profiles.

1. A. J. Kirby and R. J. Schmidt, Journal of ethnopharmacology, 1997, 56, 103–108.
2. R. Re, N. Pellegrini, A. Proteggente, A. Pannala, M. Yang and C. Rice-Evans, Free radical biology and medicine, 1999, 26, 1231–1237.
3. R. Apak, K. Güçlü, M. Özyürek and S. E. Karademir, Journal of agricultural and food chemistry, 2004, 52, 7970–7981.
4. I. F. Benzie and J. J. Strain, Analytical biochemistry, 1996, 239, 70–76.
5. P. Prieto, M. Pineda and M. Aguilar, Analytical biochemistry, 1999, 269, 337–341.
6. T. C. Dinis, V. M. Madeira and L. M. Almeida, Archives of biochemistry and biophysics, 1994, 315, 161–169.
7. G. L. Ellman, K. D. Courtney, V. Andres Jr and R. M. Featherstone, Biochemical pharmacology, 1961, 7, 88–95.
8. T. Masuda, D. Yamashita, Y. Takeda and S. Yonemori, Bioscience, biotechnology, and biochemistry, 2005, 69, 197–201.
9. I. Šafašík, Journal of Enzyme Inhibition, 1990, 3, 245–247.
10. H. Karagecili, M. A. Yılmaz, A. Ertürk, H. Kiziltas, L. Güven, S. H. Alwasel and İ. Gulcin, Molecules, 2023, 28, 1739.
